# Different Camptothecin Sensitivities in Subpopulations of Colon Cancer Cells Correlate with Expression of Different Phospho-Isoforms of Topoisomerase I with Different Activities

**DOI:** 10.3390/cancers12051240

**Published:** 2020-05-14

**Authors:** Cinzia Tesauro, Josephine Geertsen Keller, Irina Gromova, Pavel Gromov, Rikke Frøhlich, Jens Uldum Erlandsen, Anni H. Andersen, Magnus Stougaard, Birgitta R. Knudsen

**Affiliations:** 1 Department of Molecular Biology and Genetics, C. F. Møllers Allé 3, Bldg. 1131, Aarhus University, 8000 Aarhus, Denmark; ctesauro@mbg.au.dk (C.T.); jgk@clin.au.dk (J.G.K.); rff@stll.au.dk (R.F.); jens@uldum-erlandsen.dk (J.U.E.); aha@mbg.au.dk (A.H.A.); 2 Department of Clinical Medicine, Aarhus University, 8000 Aarhus, Denmark; magnstou@rm.dk; 3 Genome Integrity Unit, Danish Cancer Society Research Center, Breast Cancer Group, Strandboulevarden 49 DK, 2100 Copenhagen, Denmark; iig@cancer.dk (I.G.); psg@cancer.dk (P.G.); 4 Department of Pathology, Aarhus University Hospital, 8000 Aarhus, Denmark

**Keywords:** topoisomerase 1, colon cancer, phosphorylation, cell heterogeneity, camptothecin

## Abstract

The heterogeneity of tumor cells and the potential existence of rare cells with reduced chemotherapeutic response is expected to play a pivotal role in the development of drug resistant cancers. Herein, we utilized the colon cancer cell lines, Caco2 and DLD1, to investigate heterogeneity of topoisomerase 1 (TOP1) activity in different cell subpopulations, and the consequences for the chemotherapeutic response towards the TOP1 targeting drug, camptothecin. The cell lines consisted of two subpopulations: one (the stem-cell-like cells) divided asymmetrically, was camptothecin resistant, had a differently phosphorylated TOP1 and a lower Casein Kinase II (CKII) activity than the camptothecin sensitive non-stem-cell-like cells. The tumor suppressor p14ARF had a different effect in the two cell subpopulations. In the stem-cell-like cells, p14ARF suppressed TOP1 activity and downregulation of this factor increased the sensitivity towards camptothecin. It had the opposite effect in non-stem-cell-like cells. Since it is only the stem-cell-like cells that have tumorigenic activity our results point towards new considerations for future cancer therapy. Moreover, the data underscore the importance of considering cell-to-cell variations in the analysis of molecular processes in cell lines.

## 1. Introduction

Among the promising drugs for treatment of advanced stage colon cancers are derivatives of camptothecin (CPT) of which irinotecan is currently in clinical use [1,2]. Although treatment with irinotecan shows promising results, the development of drug resistance is still a critical problem. The cellular mechanisms underlying such developments have not been fully elucidated [3,4]. 

CPT acts via its cellular target, the enzyme human topoisomerase 1 (TOP1) [3,5,6]. TOP1 modulates DNA topology by introducing transient single stranded DNA breaks in the genome. In the absence of drugs, these breaks are rapidly sealed. However, CPT inhibits the religation step of TOP1 catalysis and thereby prolongs the half-life of TOP1-induced single stranded DNA breaks [6,7]. By collision with replication forks or transcription machinery, the breaks are converted to permanent DNA damage causing cell death. Hence, CPT converts TOP1 activity to a cell poison. Consistently, several studies in cell lines or patient samples have reported a direct correlation between TOP1 activity and/or the amount and sensitivity towards CPT [8,9,10]. However, this is not always the case and such correlations are either weak or even absent in most clinical studies [11,12,13,14]. Other molecular factors than TOP1 activity/amount, such as DNA repair, cell proliferation rate, and drug efflux activities, may affect the cellular drug response [3,8,15,16] and so may cell-to-cell variations. Indeed, the response of a few “odd one out” cells may easily be overlooked in bulk measurements. 

Compiling evidence suggests that the lack of consistency between different studies may at least in part be due to the fact that tumors and even some cell lines are characterized by a high degree of heterogeneity. Due to the uncontrolled cell division and fast growth rate of cancerous cells, even a few rare cells that survive initial treatment may determine chemotherapeutic resistance [17,18]. The heterogeneity of cancers has traditionally been attributed to clonal expansion in which different clones are generated by sequential genetic or epigenetic alterations during cancer development [19,20]. However, during the past decades it has become increasingly clear that the relatively rare cancer stem cells (CSCs) also play an important role in cancer development, progression, and chemo-resistance [21,22]. CSCs are defined as asymmetrically dividing cells giving rise to new CSCs as well as to more differentiated cells constituting the tumor bulk [23]. Moreover, they are responsible for initiating and sustaining tumor growth [24]. It is still debated whether the sequential genetic/epigenetic alterations or the existence of plastic cell subpopulations such as CSCs and non-CSCs are the major determinants of cell heterogeneity in tumors [25]. However, the two theories are not mutually exclusive [26]. It is clear that at least some cancer types and some cancer-derived cell lines are characterized by the existence of CSCs or CSC-like cell subpopulations. Characteristic for CSCs is that they can be distinguished from non-CSCs by the expression of specific cell surface markers. This allows them to be isolated and investigated. In colon cancer, the well-established markers of CSCs are CD133 and/or CD44. A selection of cell lines derived from colon cancer was reported to contain cell subpopulations with tumor initiating capacity defined by the expression of these markers. In Caco2 [27], HT29 [28], HCT116 [29], and DLD1 [30], the tumor initiating capacity of the CD133 and/or CD44 positive cells were confirmed in xenograft experiments. 

We previously used the Caco2 cell line to address the variations between CSC-like and non-CSC-like cell subpopulations within a single cell line with regard to chemotherapeutic response and molecular characteristics. As a cause of these studies we demonstrated that the CSC-like cells were insensitive towards CPT, which was consistent with these cells expressing a drug insensitive phosphor-isoform of TOP1 [31]. 

In the current study, we demonstrate that the CSC-like subpopulation of the DLD1 cell line resembles the CSC-like cells from Caco2 by being resistant towards CPT and that the resistant phenotype of both cell lines correlates to the phosphorylation state of TOP1. CPT was chosen as the representative drug for the studies since it is the mother compound of all CPT derivatives used in the clinic. Indeed, CPT and all its derivatives work by utilizing the same mechanism of action metabolic differences primarily distinguish them. We identified the protein kinase, Casein Kinase II (CKII), as an important cellular regulator of TOP1 phosphorylation and CPT response. In both the CSC-like and non-CSC-like cell subpopulations of Caco2 and DLD1, inhibition of CKII resulted in a markedly altered phosphorylation state of TOP1 and a decreased cellular CPT response. Moreover, in Caco2 we successfully demonstrated that the CPT resistance of the CSC-like cells correlated to a reduced CKII activity level. We also found that the expression of the ARF tumor suppressor protein p14ARF affected TOP1 activity and CPT sensitivity of Caco2 cells. p14ARF is a cancer associated protein that is present in low or undetectable levels in normal cells, but accumulated in cancer cells [32,33]. It has been reported to play a role in activation of the p53 tumor suppressor pathway [33,34,35]. In some cancer cells, p14ARF has also been observed to interact with and regulate the activity of TOP1 [36,37,38]. In this study, p14ARF inhibited the activity of TOP1 in the CPT-resistant CSC-like cells while it stimulated the TOP1 activity in the CPT sensitive non-CSC-like cells. Downregulation of p14ARF consequently increased the sensitivity of the CSC-like cells towards CPT treatment. 

## 2. Results

### 2.1. Colon Cancer Cell Lines Can Be Divided into Subpopulations Based on the Expression of Cell Surface Markers

In the current study, we aim to investigate how molecular characteristics of CSC-like cells contribute to the chemotherapeutic response of colon cancer cell populations. To do so it is essential to identify and separate such cell subpopulations from the bulk. As mentioned in the introduction, the expression of CD133 and CD44 are well-characterized markers of CSC-like cells in colon cancer [27,39]. Consequently, we analyzed the colon cancer derived cell lines Caco2 and DLD1 (Figure S1), as well as SW480 and HT29 for the existence of cell subpopulations with different expression of these markers. As indicators of CSC-like features we measured the sensitivity of the subpopulations towards NaBt-induced differentiation [27,40] and their ability to divide asymmetrically [41,42]; both are characteristics of CSC-like cells with tumor initiating capacity [41,42,43]. Of the examined cell lines, only Caco2 and DLD1 contained a NaBt sensitive cell subpopulation with sufficient abundancy (more than 5%) for analysis. As evident from Figure S1, we found that the CD133 positive cells of DLD1 and the CD44 positive cells of Caco2 cell subpopulations behaved similarly and both demonstrated sensitivity towards NaBt treatment (Figure S1A,B) and asymmetric cell division over time (Figure S1C,D). This is also in agreement with the tumor initiating capacity of these cell subpopulations as reported elsewhere [30,44,45]. Consequently, the CD133 positive DLD1 and the CD44 positive Caco2 subpopulations will be referred to as CSC-like cells in the following sections. 

### 2.2. The Cancer Stem-Cell-Like Cell Subpopulations of Colon Cancer Derived Cells Survive Camptothecin Treatment

We previously demonstrated that the CSC-like cells of Caco2 were CPT resistant. The resistance correlated to a decreased activity of the intracellular TOP1 that at least in part depended on the phosphorylation state of TOP1 [31]. As evident from Figures S2 and S3, the CSC-like DLD1 cells also exhibited a CPT-resistant phenotype that correlated to a low TOP1 activity. This reduced activity may be partly explained by a low expression level (see Figure S3A) and partly by an altered phosphorylation state of TOP1 in the CSC-like relative to the non-CSC-like cells (Figure S3C–E). 

**Figure 1:**
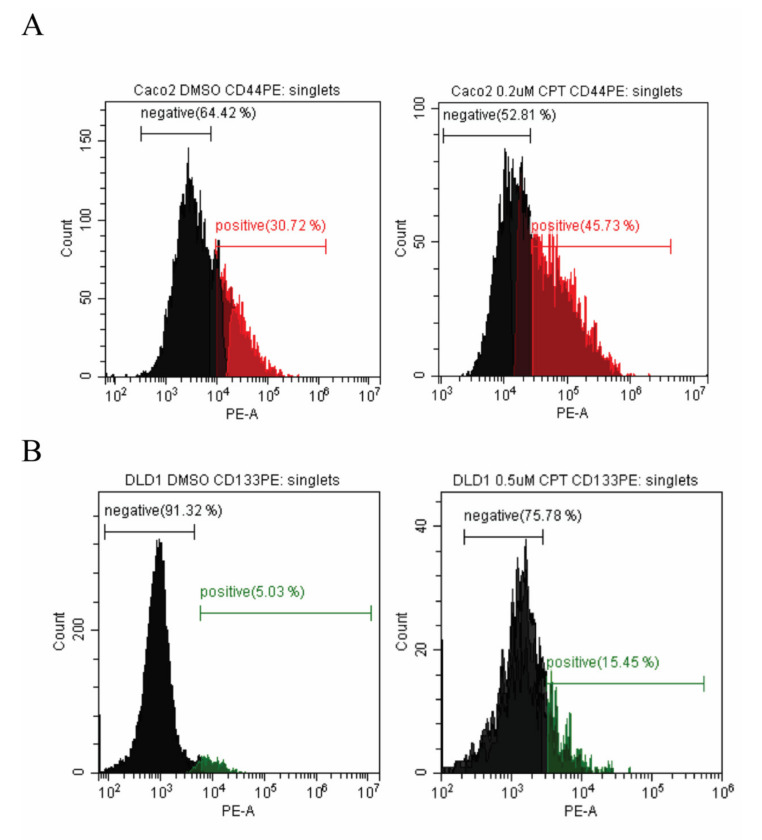
Flow-cytometry analysis of Caco2 and DLD1 before and after camptothecin treatment. The cells were treated with DMSO (**A** and **B**, left panels) or indicated concentrations of camptothecin (CPT) (**A** and **B**, right panels) for 72 h before labeling and flow-cytometry analysis. Cancer stem cell (CSC)-like Caco2 cells are shown in red (**A**) and CSC-like DLD1 cells are shown in green (**B**).

To investigate how CPT treatment affected the composition of colon cancer cell populations, unsorted cells of Caco2 or DLD1 were treated with or without CPT (concentrations indicated in Figure 1). After 72 h of treatment the percentages of CSC-like or non-CSC-like cells in the surviving population were determined by flow-cytometry analyses. As evident from the histogram shown in Figure 1, the size of the CSC-like cell fraction increased (from 31% to 46% for Caco2 and from 5% to 15% for DLD1) in the CPT-treated cell cultures. This result implies that CSC-like cells may play a role in drug resistance in colon cancer. Note that given the asymmetric cell division capability of CSC-like cells, the surviving cells will be able to restore cell line heterogeneity. 

### 2.3. CKII Modulates the Camptothecin Response of Colon Cancer Cells via Phosphorylation of Topoisomerase 1

Previous in vitro investigations using purified components demonstrated that the CKII kinase may be a player in the regulation of TOP1 activity [31,46]. It is therefore likely that CKII kinase activity may be an important determinant of the CPT response of the different cell subpopulations of colon cancer cells. 

To investigate this possibility, DLD1 and Caco2 cells were cultivated in the absence or presence of the highly specific CKII inhibitor 4,5,6,7-tetrabromobenzotriazole (TBB) [47,48] before they were sorted by Fluorescence Activated Cell Sorting (FACS) and the cellular CPT response of the isolated cell subpopulations measured. The inhibitory effect of TBB was confirmed to approximately 50% reduction of kinase activity in both cell lines (Figure S4). 

**Figure 2:**
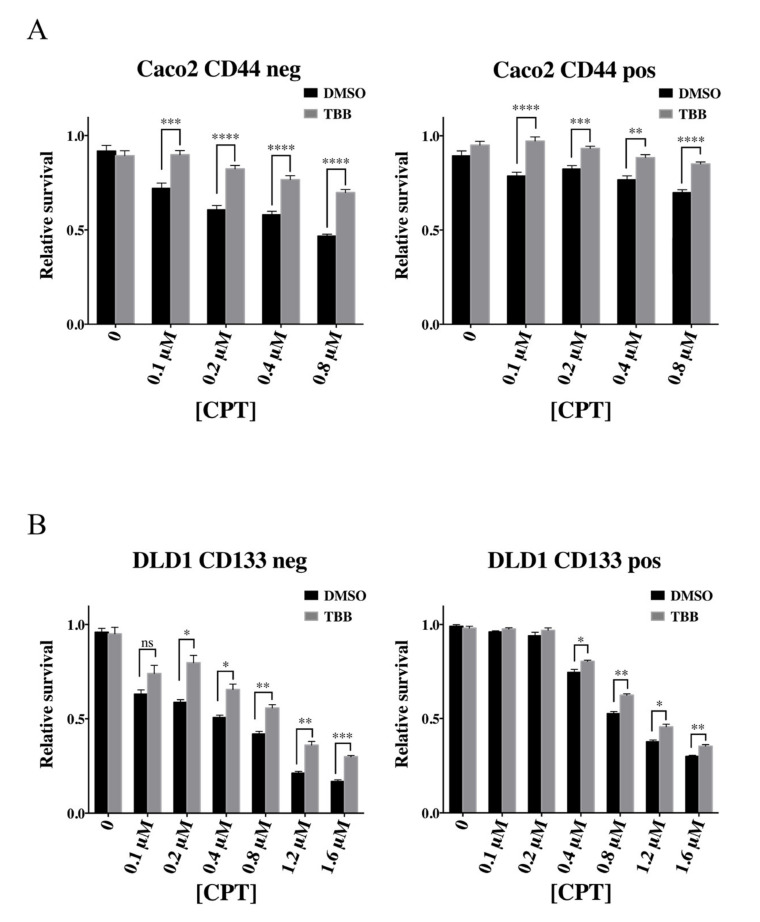
Camptothecin sensitivity of colon cancer cell subpopulations with or without treatment with 4,5,6,7-tetrabromobenzotriazole (TBB). (**A**) Survival assay of the Fluorescence Activated Cell Sorting (FACS) sorted non-CSC-like (CD44 negative, “CD44 neg”, left graph) or CSC-like (CD44 positive, “CD44 pos”, right graph) cells of Caco2 after treatment with DMSO or 10 μM TBB. The sorted cells were plated and treated with DMSO or indicated concentrations of CPT for 72 h before the percentage of viable cells was measured using the PrestoBlue viability assay. Data were normalized to the viability of the cells without CPT (DMSO) and plotted as mean +/− standard error of the mean (SEM). **** *p* < 0.0001, *** *p* < 0.001; ** *p* < 0.01, Welch’s *t*-test, *n* = 6. (**B**) Same as (A) except that DLD1 cell subpopulations were analyzed. *** *p* < 0.001; ** *p* < 0.01; * *p* < 0.05, Welch’s *t*-test.

The CPT response of the sorted cell subpopulations was measured using a standard PrestoBlue cell viability assay after treatment with CPT concentrations as indicated in Figure 2. For subpopulations of both cell lines, TBB treatment reduced the cellular CPT response significantly (compare the grey and black bars of all panels in Figure 2). These results strongly suggest that CKII plays an important role for CPT response of colon cancer derived cell lines. 

For Caco2, we confirmed that the reduced CPT response of both the TBB-treated CSC-like and non-CSC-like cells correlated with a reduced phosphorylation of the intracellular TOP1 (see Figure S5A,B). TBB treatment did not affect the protein level of TOP1 in neither of the cell subpopulations (Figure S5C). The reduced phosphorylation of TOP1 was accompanied by a reduced activity level of the enzyme (Figure S5D). This result is consistent with previous observations obtained in studies using purified components [31]. Moreover, the obtained result lends further credence to the notion that the CPT sensitivity of colon cancer cells is highly influenced by the intracellular CKII activity and that this effect at least partly can be explained by altered TOP1 activity. Note that similar experiments were not performed in DLD1 due to insufficient abundancy of CSC-like cells in this cell line. 

### 2.4. The Cancer Stem-Cell-Like Subpopulation of Caco2 Cells Is Characterized by a Low CKII Kinase Activity

**Figure 3:**
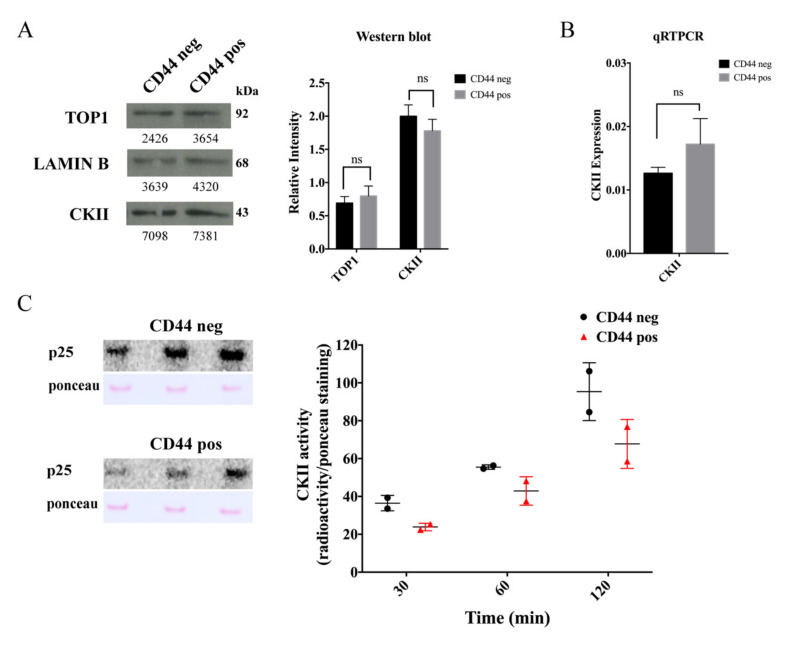
Assessment of CKII in cancer stem-cell-like and non-cancer stem-cell-like Caco2 cells. (**A**) Left panel, Western blot analysis of nuclear extracts from non-CSC-like (CD44 negative, “CD44 neg”) or CSC-like (CD44 positive, “CD44 pos”) cells, developed using an anti-CKII antibody, anti-topoisomerase 1 (TOP1) antibody, or anti-LAMIN B antibody (as a loading control). Right panel, graphical depiction showing the results of densitometrical quantification of the bands shown in the Western blot. The intensity of the TOP1 and CKII specific bands were normalized relative to the intensity of the LAMIN B bands. Data were plotted as mean +/− standard error of the mean (SEM). The whole western blot figures are in Figure S10A. (**B**) Graphical depiction of the results for qRT-PCR analysis of CKII mRNA. CKII gene expression is calculated based on the Ct values using the following equations: ∆Ct = Ct(target) − Ct(reference) and ∆Ct^exp^ = 2^−∆Ct^. Data were plotted as mean +/− standard error of the mean (SEM). ns: not significative difference (**C**) Left panels, CKII activity measured in nuclear extracts from non-CSC-like Caco2 cells (CD44 negative, “CD44 neg”) or CSC-like Caco2 cells (CD44 positive, “CD44 pos”). p25: representative autoradiogram of a membrane with p25 substrate (purified TOP1, amino acids (a.a.) 1–206), incubated with the nuclear extract from non-CSC-like Caco2 cells (CD44 negative) or Caco2 CSC-like cells (CD44 positive), γ-^32^P-ATP and inhibitors of non-CKII kinases for 30, 60, and 120 min. Ponceau: Ponceau stain of the same membranes. Right panel, graphical depiction of the ratio between the densitometric quantification of the radiolabeled bands (phosphorylated p25) and the intensity of the Ponceau-stained p25 for the indicated time intervals. The graph shows values of two independent experiments.

To investigate if different CKII activities could explain the different CPT response of CSC-like versus non-CSC-like cells, the CKII status in the two Caco2 cell subpopulations was addressed. This was done at the expression and activity levels. Expression was measured at the mRNA level using qRT-PCR and at the protein level by Western blotting using an anti-CKII antibody. As evident from Figure 3A,B, expression of CKII was comparable in the two cell subpopulations both at the mRNA and protein levels. The CKII activity level in extracts from either CSC-like or non-CSC-like cells was measured by the phosphorylation of a TOP1 derived marker peptide (p25) encompassing amino acids 1–206 [49] using ^32^P-γATP as the phosphoryl donor (see Section 4). The CKII activity was higher in extracts from non-CSC-like relative to CSC-like cells as evident when comparing the intensity of the auto-radiographically detected bands representing the phosphorylated marker peptide in Figure 3C. This result implies that the CPT-resistant phenotype of the CSC-like Caco2 cell subpopulation may at least in part be explained by a lower CKII activity, which in turn affects the phosphorylation state and activity of TOP1. Note that the abundancy of DLD1 CSC-like cells was too low for the CKII status to be measured in these cells. 

### 2.5. The Tumor Suppressor p14ARF Affects the Topoisomerase 1 Activity and Camptothecin Sensitivity Differently in Cancer Stem-Cell-Like and Non-Cancer Stem-Cell-Like Cells

**Figure 4:**
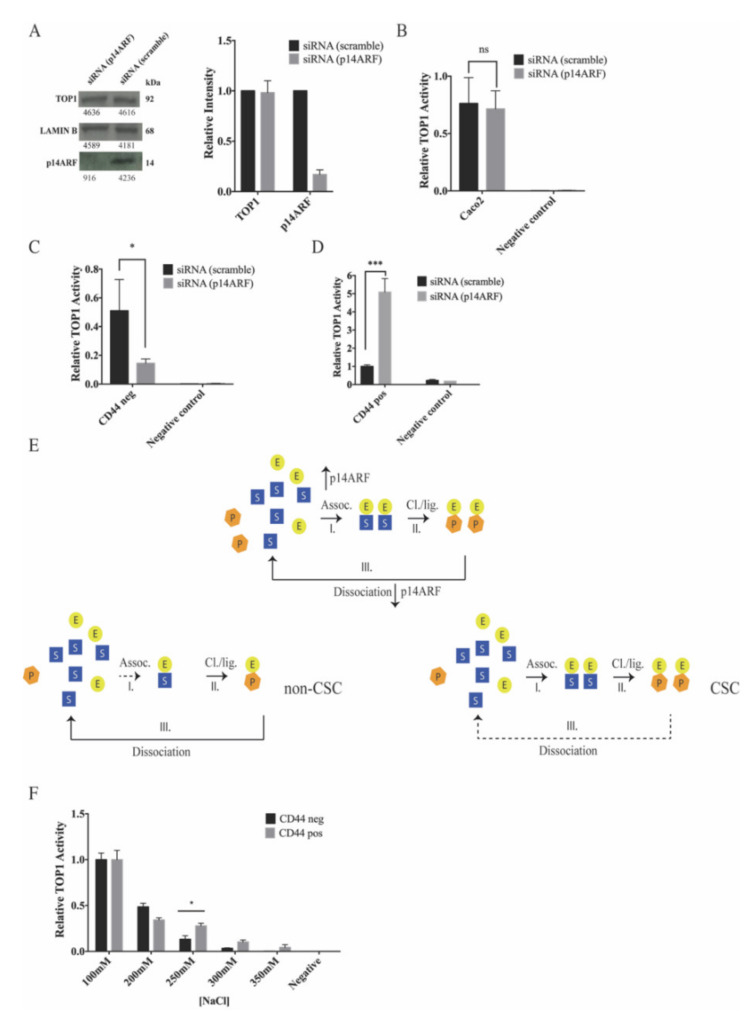
Effect of p14ARF downregulation on topoisomerase 1 activity. (**A**) Left panel, Western blot analysis of whole cell extracts from unsorted Caco2 cells transfected with scrambled siRNA (siRNA (scramble)) or p14ARF specific siRNA (siRNA (p14ARF)) developed by using anti-TOP1, anti-p14ARF, or anti-LAMIN B antibodies. Right panel, graphical depiction showing the results of the densitometrically quantified bands shown in the Western blot. The intensity of the p14ARF or TOP1 specific bands was normalized relative to the intensity of the LAMIN B bands. Data were plotted as mean +/− standard error of the mean (SEM). (**B**) Measurement of TOP1 activity by rolling-circle enhanced enzyme activity detection (REEAD) in whole cell extracts from Caco2 cells transfected with siRNA (scramble) (black bars) or siRNA (p14ARF) (grey bars). The REEAD signals detected on an average of 12 microscopic images were counted using the ImageJ software and the result was normalized against the number of signals obtained by analyzing the activity of purified TOP1. The signals were normalized as reported by Stougaard et al. [50]. All data were plotted as mean +/− SEM, ns = not significative, Welch’s test, *n* = 6. (**C**) Same as (**B**), except that non-CSC-like cells (CD44 negative) were analyzed. The non-CSC-like cells were obtained by treating the cells with NaBt, giving approx. 100% CD44 negative cells. The data were plotted as mean +/− SEM. * *p* = 0.03, Welch’s test, *n* = 6. (**D**) Measurement of TOP1 activity in the whole cell extracts from Caco2 CSC-like (CD44 positive) cells transfected with siRNA (scramble) (black bars) or siRNA (p14ARF) (grey bars). The CSC-like (CD44 positive) cells were captured onto a glass slide by using anti-CD44 antibody and the TOP1 activity measured by using the On-Slide-REEAD as described by Keller et al. [51]. The REEAD signals were counted using the ImageJ software and the result was normalized against the number of signals obtained by analyzing the activity of purified TOP1. The signals were normalized as reported by Andersen et al. [52]. The data were plotted as mean +/− SEM. *** *p* = 0.0002, Welch’s test, *n* = 6. (**E**) Schematic illustration of the catalytic steps that determine the reaction rate of TOP1. First, the enzyme (yellow circle, E) associates (I) with the substrate (blue square, S) to form a non-covalent binding complex. Thereafter, the enzyme performs cleavage–ligation (II) to generate a product (orange hexagon, P) still associated with the enzyme. Finally, the enzyme dissociates (III) from the product and is ready to perform another round of catalysis. p14ARF stimulates non-covalent DNA binding. Thereby it stimulates association and inhibits dissociation (illustrated by arrows pointing up for stimulation and down for inhibition). The lower left panel illustrates how a weakened association in non-CSC cells will affect activity while the lower right panel illustrates how a weakened dissociation in CSC cells will affect activity. (**F**) Measurement of TOP1 activity in the nuclear extracts from Caco2 non-CSC-like (CD44 negative) (black bars) and Caco2 CSC-like (CD44 positive) (grey bars) FACS sorted cell subpopulations, respectively. The activity was measured by REEAD at different NaCl concentrations as reported on the x-axis. The REEAD signals were counted using the ImageJ software and the result was normalized against the number of signals obtained by analyzing the activity of purified TOP1. All data were plotted as mean +/− SEM. * *p* < 0.04, Welch’s *t*-test. The whole western blot figures please find in Figure S10B.

It was previously demonstrated that the tumor suppressor p14ARF can interact with and regulate the activity of phosphorylated isoforms of TOP1 and thereby affect the CPT sensitivity in cancer cells [36,37,38]. As evident in Figure S6, both subpopulations of Caco2 expressed p14ARF to a comparable level. To investigate whether p14ARF plays a role in regulating the activity of TOP1 in these cells, Caco2 cells were transfected with p14ARF specific or scrambled siRNA. Knockdown of p14ARF in cells treated with p14ARF specific siRNA was confirmed to be approximately 80% by Western blotting (Figure 4A). 

The effect of downregulating p14ARF on TOP1 activity was investigated by the previously described rolling-circle enhanced enzyme activity detection (REEAD) assay [31,50] using extracts from a mixed cell population as well as from non-CSC-like or CSC-like cells (Figure 4B–D). For the mixed cell population, whole cell extracts were prepared from the siRNA (scramble) or siRNA (p14ARF) transfected Caco2 cells and TOP1 activity in these extracts measured by REEAD. To measure the effect of p14ARF on TOP1 activity in non-CSC-like cells, cell differentiation was induced by NaBt treatment (generating approximately 100% non-CSC-like cells, Figure S1A,B) subsequent to siRNA transfection. Thereafter, TOP1 activity was measured by REEAD in whole cell extracts. Measurement of TOP1 activity in the CSC-like cell subpopulation was achieved by trapping the cells on a glass slide based on CD44 expression before they were lysed and the TOP1 activity measured using the On-Slide-REEAD assay as previously described [51]. As evident from the graphical depiction of the results in Figure 4B–D, p14ARF knockdown had little effect on the average TOP1 activity in extracts from the mixed population of Caco2 cells, while p14ARF knockdown decreased TOP1 activity in the non-CSC-like cells more than three-fold and increased the activity in the CSC-like subpopulations around five-fold. These results highlight the value of investigating each cell subpopulation separately rather than doing bulk analysis on the entire cell population. Note that due to the limited number of siRNA transfected cells available to the investigation, advantage could not be taken from FACS sorted cells for these experiments. Hence, when assaying the TOP1 activity in mixed or non-CSC-like subpopulations versus CSC-like cells, it was necessary to use different assay setups (standard REEAD versus On-Slide-REEAD). This made it impossible to use equal numbers of cells in all experiments. The REEAD signals counted in each experiment (mixed-, non-CSC-like, or CSC-like cell populations) were therefore normalized as previously reported [52]. This normalization was appropriate since the purpose of the experiment was to measure the effect of p14ARF on TOP1 activity within each cell (sub)population (raw data are shown in Figure S7). 

The different effect of p14ARF on TOP1 in CSC-like versus non-CSC-like cells could be the result of different DNA binding affinities of TOP1 in the two cell subpopulations. This theory is fostered by the fact that p14ARF previously stimulated DNA binding of TOP1 without affecting the cleavage–ligation rate [37]. If the cleavage–ligation activity is kept constant, and the DNA substrate is in molar excess (as is the case in our assay setup), the activity of TOP1 as measured in the REEAD assay is determined by the balance between DNA association and dissociation only. Inhibition of DNA association will hamper TOP1 binding and the subsequent cleavage step of catalysis, which will result in less REEAD signals [50]. Inhibition of DNA dissociation will prevent the enzyme from leaving one substrate and react with a new one and will also result in less REEAD signals (see schematic in Figure 4E). Based on these considerations, the observed inhibition of TOP1 by p14ARF knockdown in non-CSC-like cells may be the result of a relatively low DNA affinity of TOP1 resulting in DNA association being the rate limiting step of catalysis. The observed stimulation of TOP1 by p14ARF knockdown in the CSC-like cells may indicate a relatively high DNA affinity of TOP1 resulting in DNA dissociation being the rate limiting step of catalysis. To investigate these possibilities, the DNA affinity of TOP1 in extracts from the two cell subpopulations was addressed in terms of NaCl tolerance using the REEAD assay. Note that NaCl inhibits the non-covalent DNA binding of TOP1 [49,53]. Hence, a higher NaCl tolerance is indicative of a higher DNA binding affinity of the enzyme. As shown in Figure 4F, TOP1 in whole cell extracts from CSC-like cells was significantly more NaCl tolerant than TOP1 in extracts from non-CSC-like cells (compare the grey and black bars at and above 250 mM NaCl). This result is consistent with TOP1 in CSC-like cells having a higher DNA affinity than TOP1 in non-CSC-like cells, which in turn may explain the different effect of p14ARF on TOP1 activity in the two cell subpopulations. Unfortunately, due to technical limitations it was not possible to measure the NaCl tolerance in the two cell subpopulations after p14ARF knockdown. 

### 2.6. p14ARF Knockdown Increases the Camptothecin Response of Cancer Stem-Cell-Like Caco2 Cells and Decreases the Camptothecin Response of Cancer Non-Stem-Cell-Like Caco2 Cells

The enhanced TOP1 activity in CSC-like cells after p14ARF knockdown suggests that reducing p14ARF expression may enhance CPT response of these cells. To investigate this possibility Caco2 cells were treated with scrambled or p14ARF specific siRNA followed by treatment with 0.5 μM CPT or DMSO as a control. Subsequently, the cells where stained with propidium iodide (PI; as a marker for dead cells) and an allophycocyanin (APC) conjugated anti-CD44 antibody and analyzed for dead versus alive CD44 positive or negative cells by flow-cytometry. The results are shown as dot plots in Figure S8 and summarized in Table 1. As evident from the table, the percentage of dead CD44 positive (CSC-like) cells induced by CPT treatment increased from 25.3% to 38% upon p14ARF knockdown. In contrast, p14ARF knockdown reduced the percentage of CD44 negative (non-CSC-like) dead cells induced by CPT from 74.7% to 62%. This result is in agreement with the increased TOP1 activity in the CSC-like cells and the reduced TOP1 activity in non-CSC-like cells upon p14ARF knockdown. It suggests that the CSC-like cells are sensitized towards CPT upon p14ARF downregulation. Consistently, p14ARF knockdown prevented accumulation of CSC-like cells upon CPT treatment (see Figure S9). Since the CSC-like cells have tumorigenic capacity, it is of particular importance to target these cells. The presented results suggest that this may be accomplished by combining CPT treatment with p14ARF downregulation. As evident from Table 1, p14ARF knockdown alone induced cell killing in Caco2. Since p14ARF is a cancer specific protein that is present at low to undetectable levels in normal cells [32,37] it is unlikely that p14ARF knockdown will affect normal human cells. 

## 3. Discussion

In the present study, we demonstrated the existence of dynamic cell subpopulations with different CPT sensitivity in colon cancer derived cell lines DLD1 and Caco2. These cell subpopulations could be distinguished on the basis of expression of CD133 and CD44 that are well established markers for stemness characteristics in colon cancer [27,39,45]. Consistently the CD133 positive cells of DLD1 and the CD44 positive cells of Caco2 demonstrated asymmetric cell division and were sensitive towards NaBt-induced differentiation in accordance with cells having CSC-like characteristics. 

For both cell lines, we observed that the asymmetrically dividing CSC-like cell subpopulations were resistant towards CPT treatment and were enriched upon CPT treatment. This could indicate that CSC-like cells may be an important source of CPT resistance in colon cancer. 

For Caco2, the CPT resistance was previously reported to correlate with a relatively low activity of TOP1 [31]. In the present study, we demonstrated a similar correlation between low cellular CPT sensitivity and low TOP1 activity in the CSC-like DLD1 cell subpopulation. For both cell lines, the differential TOP1 activity levels and CPT responses of the investigated cell subpopulations corresponded to different phosphorylation states of TOP1 as demonstrated by 2D gel analyses. 

Protein kinase CKII previously played a key role in phosphorylation and regulation of TOP1 in a large number of cancer cell lines [46,48,54]. This was implicated in the regulation of TOP1 activity in the different Caco2 cell subpopulations by in vitro investigations using cell extracts treated with or without purified CKII or phosphatases [31]. In the present study, we addressed the in vivo role of CKII in the regulation of the CPT response in Caco2 and DLD1 cells by investigating the cellular effect of CKII inhibition by TBB. We found that TBB treatment decreased the CPT sensitivity of both the CSC-like and the non-CSC-like cell subpopulations in the two cell lines. These results strongly argue for the involvement of CKII in regulation of the cellular CPT response of colon cancer derived cell subpopulations. In Caco2, it was confirmed that the decreased CPT sensitivity of the TBB-treated cells correlated with a reduced phosphorylation and activity of TOP1. In the DLD1 cell line, the number of CSC-like cells was too low for such investigations to be completed. In agreement with our previous work, we also demonstrated that the CPT resistance of the CSC-like Caco2 cells relative to non-CSC-like cells correlated with a decreased TOP1 activity level and an altered phosphorylation state of TOP1. We furthermore demonstrated that the CSC-like Caco2 cell subpopulation was characterized by low CKII activity. 

Our data are consistent with the compelling evidence of phosphorylation being critical for TOP1 activity and CPT sensitivity [55,56,57,58] and of CKII playing a central role in this regulation [46,48,54]. Moreover, the differential CPT response of CSC-like versus non-CSC-like colon cancer cells may at least in part be ascribed to different intracellular CKII activity levels, which in turn affect TOP1 activity levels. 

It was previously demonstrated by the Gjerset group that the tumor suppressor p14ARF can interact with and stimulate DNA binding of certain phosphor isoforms of TOP1 [36,37]. Such interaction was demonstrated to modulate TOP1 activity and CPT sensitivity of a broad range of cancer cell lines [37]. Spurred on by these findings we set out to address a potential interplay between TOP1 and p14ARF in the two cell subpopulations of Caco2. We found that p14ARF affected TOP1 activity differently in the CSC-like and non-CSC-like cells. In the CSC-like cells, TOP1 activity was increased, and in the non-CSC-like cells TOP1 activity was decreased upon p14ARF downregulation. In contrast, we did not observe any significant change in TOP1 activity in extracts from mixed Caco2 cells upon p14ARF downregulation. This is most likely due to the increased activity in one cell subpopulation counteracting the decreased activity in the other. The increased activity of TOP1 in CSC-like cells upon p14ARF downregulation correlated with a high salt tolerance of TOP1 in extracts from CSC-like cells relative to TOP1 in extracts from non-CSC-like cells. A high salt tolerance of TOP1 is indicative of a high DNA binding affinity and a corresponding low DNA dissociation rate [49,53]. It was previously demonstrated that p14ARF stimulates TOP1 DNA binding without affecting the cleavage–ligation rate. If the cleavage–ligation rate is kept constant, the activity of TOP1 in a catalytic assay (as the utilized REEAD assay) will depend on a combination of DNA association and dissociation. The DNA association should be sufficiently high to allow for efficient binding, and yet low enough to allow for the enzyme to dissociate from one DNA molecule and act on a new one (see also Figure 4E). Hence, both too low and too high DNA binding (that affects substrate association and dissociation) of TOP1 will have an inhibitory effect on the catalytic activity of the enzyme. Taking these considerations into account, the increased TOP1 activity in CSC-like Caco2 cells upon p14ARF knockdown may be explained by “higher-than-optimal” DNA affinity of TOP1 in these cells. When the p14ARF level is reduced, the DNA affinity may be lowered sufficiently to allow for efficient enzyme turnover and dissociation, thereby increasing the rate by which the enzyme can act on a new DNA substrate. The activity of TOP1 in extracts from the non-CSC-like Caco2 cells was less salt tolerant than the activity of TOP1 in extracts from the CSC-like cells. This could indicate a lower binding affinity which in turn could explain the negative effect of p14ARF downregulation on TOP1 activity in these cells. Recall that p14ARF stimulated DNA binding. Hence, downregulation of p14ARF may simply result in a “lower-than-optimal” DNA affinity of TOP1 in the non-CSC-like cell subpopulation. 

Interestingly the regulatory effect of p14ARF on TOP1 in the two cell subpopulations of Caco2 was directly reflected in the cellular CPT sensitivity. p14ARF knockdown increased the CPT sensitivity of CSC-like cells relative to non-CSC-like cells and prevented accumulation of CSC-like cells after CPT treatment. Since CSCs have tumorigenic capability and are the major contributors to tumor recurrence and development of resistance, it is important to identify new possibilities for targeting this cell subpopulation of cancer. We believe that the findings presented in the current study may pave the road for the development of improved treatment protocols in the future by combining CPT derivatives with p14ARF targeting drugs. On top of the synergistic effect of p14ARF downregulation with CPT (resulting in a total of 45% dead cells at the utilized condition), p14ARF knockdown by itself also induces a substantial cell killing (resulting in a total of approximately 32% dead cells). Whether such an effect is specific for cancer cells or may result in unwanted side effects remains to be addressed. However, since p14ARF expression is rather specific for cancer cells we believe the potential effect of p14ARF knockdown on non-cancer cells to be modest. 

## 4. Materials and Methods

### 4.1. Reagents, Antibodies, and Enzymes

Human anti-CD44-PE Antibody, human anti-CD133-PE Antibody, and FcR Blocking Reagent were purchased from MACS Miltenyi Biotech (Bergisch Gladbach, Germany). Mouse anti-CKII (#sc-373894) and mouse anti-LAMIN B (#sc-365214) were purchased from Santa Cruz Biotechnology (Dallas, TX, USA), rabbit anti-p14ARF (#SAB4500073) was from Sigma-Aldrich ApS (Søborg, Denmark) and human anti-TOP1 (#TG2012) was from TopoGEN (Buena Vista, CO, USA). Horse radish peroxidase, HRP conjugated goat anti-mouse, goat anti-human, and goat anti-rabbit were all purchased from DAKO (ThermoFisher Scientific, Roskilde, Denmark). Phi29 DNA polymerase and PrestoBlue reagent were purchased from Thermo Fisher Scientific (Roskilde, Denmark). All chemicals, cells media, and media components were purchased from Sigma-Aldrich ApS (Søborg, Denmark). α^32^P-dATP was purchased from PerkinElmer (Skovlunde, Denmark). 

### 4.2. Cells Cultures

All cell lines were obtained from the American Type Culture Collection ATCC and grown in Minimal Essential Medium (MEM) supplemented with 20% fetal bovine serum (FBS), 1% non-essential amino acids (NEAA), 100 units/mL penicillin, and 100 mg/mL streptomycin (Sigma-Aldrich ApS, Søborg, Denmark). DLD1 cells were a kind gift from Professor Lene Nejsum of Aarhus University (Aarhus, Denmark). 

The cell cultures were maintained in a humidified incubator (5% CO_2_/95% air atmosphere at 37 °C). Cells were plated into tissue culture flasks (Nunc, Roskilde, Denmark) and split every 3 days to maintain them at 70% confluency. Cells were harvested by trypsin treatment (0.1% Trypsin-EDTA solution, Sigma-Aldrich ApS, Søborg, Denmark) followed by two consecutive washes with 1 × PBS and stored at −80 °C as dried pellets until further analysis. All cell lines were genetically authenticated by the ATCC and mycoplasma tested by Eurofins Genomics (GATC service, Eurofins Genomics Ebersberg, Germany). Results of mycoplasma analysis were negative. 

### 4.3. Flow-Cytometry Analysis and Cell Sorting

The identification and the validation of the cell surface markers were carried out with NaBt treatment [27]. Caco2 and DLD1 cells were seeded to a concentration of 10^6^ cells in a T175 cm^2^ flask and after 24 h the cells were treated with 5 mM NaBt or ddH_2_O. After 72 h of treatment, the cells were harvested and analyzed by flow-cytometry. When reported, the cells were treated for 72 h with indicated concentrations of CPT or with 10 μM TBB (followed by CPT) before flow-cytometric analysis. 

For flow-cytometry analysis the Caco2 and DLD1 cell lines were harvested by trypsin treatment: 0.25% trypsin and 0.02% EDTA (Sigma-Aldrich ApS, Søborg, Denmark). After resuspension of the cells in cell-culture media, the cells were counted using a Bürker–Türk chamber (Sigma-Aldrich ApS, Søborg, Denmark), washed in PBS with 0.5% Bovine Serum Albumin BSA and collected by centrifugation. The cells were incubated for 30 min in blocking buffer (1 × PBS, 1% BSA, 2.5 mM EDTA, 25 mM HEPES, 20% FcR blocking reagent) at 4 °C and stained for 30 min at 4 °C with the fluorochrome conjugated antibodies CD44-PE or CD133-PE following the manufacturer’s instructions. Following the antibody labeling the cells were washed in PBS with 0.5% BSA before flow-cytometric analysis that was carried out on a CytoFLEX (Beckman Coulter, Copenaghen, Denmark) instrument. Dying and dead cells were stained with 1 μg/mL propidium iodide (PI) and excluded from analysis. PE and PI were excited with a 561 nm laser and fluorescence emitted was collected with a 585/42 band pass filter. Doublets and cell debris were excluded by gating with FSC and SSC. CytExpert software (Beckman Coulter, Copenaghen, Denmark) was used to perform the analysis. Compensation analysis was performed. 

For cell sorting, after labeling, the cells were kept at 4 °C, filtered with a cell strainer (50 µm) and sorted using a FACSAria III (BD Biosciences, San Jose, CA, USA) with a 561 nm laser to excite both PE and PI. Light emitted from PE was collected using a 582/15 band pass filter while light emitted from PI was collected using a 610/20 band pass filter. Doublets and dead cells were excluded and Caco2 CD44 negative and CD44 positive cells and the DLD1 CD133 negative and CD133 positive cells were sorted. 

For each experiment, the background fluorescence was measured using both un-labeled cell mixture and cell mixture labeled with the appropriate concentration of PE-conjugated antibodies. After each FACS sorting the enriched fractions were analyzed for CD44 positive and CD133 positive cell purity. 

### 4.4. Cell Survival Assay

Cell survival assays of Caco2 or DLD1 cells with and without drug treatments were performed by using the PrestoBlue cell viability reagent following the manufacturer’s instructions. A 20 mM stock of CPT and 5 mM stock of TBB were prepared in 99.9% DMSO and all the working dilutions were prepared in the cell media. Then, 500 cells/100 μL media were plated into 96-well flat bottom plates (Corning, Inc., Corning, NY, USA) and incubated for 24 h. Cells were treated with DMSO or 10 μM TBB and after 48 h the media was replaced with medium containing DMSO or different concentrations of CPT ranging from 0.1 μM to 0.8 μM or from 0.1 μM to 1.6 μM for the Caco2 or DLD1, respectively, and further incubated for 72 h. At the end of the 72 h of treatment, 10 μL of PrestoBlue reagent was added and the samples incubated for 4 h at 37 °C. The fluorescence emitted was measured (540 nm excitation/590 nm emissions) using a FLUOstar OPTIMA microplate reader (BMG Labtech, Ortenberg, Germany). DMSO concentration was corrected to 0.5% in all wells. DMSO-treated cells were considered as 100% viable. Data were plotted as mean (12 wells) with standard error of the mean (SEM). Values were plotted using GraphPad Prism software. 

### 4.5. Preparation of Nuclear Cell Extract

Cells were harvested by treatment with 0.05% trypsin solution (Sigma-Aldrich, Søborg, Denmark A/S) counted with a Bürker–Türk chamber (Sigma-Aldrich, Søborg, Denmark A/S) and the pellets stored at −80 °C. For preparation of nuclear cell extracts cells were lysed in lysis buffer (0.1% NP-40, 10 mM Tris-HCl, pH 7.9, 10 mM MgCl_2_, 15 mM NaCl, 0.1 mM phenylmethylsulfonyl fluoride (PMSF), 1 mM beta glycerophosphate, 19 mM sodium fluoride (NaFl) and Roche proteases and phosphatases inhibitors cocktail, EDTA free) at 4 °C for 10 min. Subsequently, the nuclei were pelleted by centrifugation at 400× *g* for 10 min. The pelleted nuclei were extracted by addition of 100 μL nuclear extraction buffer (0.5 M NaCl, 20 mM HEPES, pH 7.9, 20% glycerol, 0.1 mM PMSF, 1 mM beta glycerophosphate, 19 mM NaFl and Roche proteases and phosphatases inhibitors cocktail, EDTA free) followed by rotation for 1 h at 4 °C [59]; fresh PMSF was added every 15 min. Cell debris were removed by centrifugation at 9000× *g* for 10 min at 4 °C and the nuclear extracts collected into a new tube and kept at 4 °C for further analysis. 

### 4.6. CKII Activity

The activity of CKII in nuclear extracts was measured using the Millipore Casein Kinase 2 Assay Kit (#17-132, Millipore, Darmstadt, Germany). The Glutathione S-transferase (GST) tagged N-terminal domain of TOP1 (a.a. 1–206) (p25) was used as substrate and purified as described previously [49]. Nuclear extracts from 10^7^ cells were normalized using Bradford quantification and incubated with the substrate in the buffer provided by the kit and 12.5 mCurie/ml α-^32^P-dATP. The reactions were incubated at 30 °C for different time intervals and the reactions stopped with the addition of 0.5% SDS. The proteins were run on a 10% SDS gel in 25 mM Tris-HCl pH 8.6, 192 mM glycine, 0.1% SDS for 1 h at 50 mA constant. The proteins were transferred onto a nitrocellulose membrane using a wet blotting apparatus for 16 h at 30 V constant in a 20 mM CAPS pH 10 and 20% ethanol at 4 °C. The membranes were exposed in a phosphorimager cassette (Perkin Helmer, Skovlunde, Denmark) for 16 h. The intensities of the radioactive bands were quantified using QuantityOne software (Bio-Rad, Copenaghen, Denmark). The membranes were stained with reactions. 

### 4.7. Western Blot

Whole cell extraction was prepared by lysing a 10^6^ cell pellet in 100 µL lysis buffer (1 mM Tris-HCl pH 7.5, 0.1 mM EDTA supplemented with PMSF, 19 mM NaFl, 1 mM beta glycerophosphate and Roche proteases and phosphatases inhibitors cocktail, EDTA free). The cells were lysed for 10 minutes in ice and the cells extracts were harvested by centrifugation at 9000× *g* at 4 °C for 10 min. Extracts were mixed with SDS loading buffer (250 mM Tris-HCl pH 6.8, 10% SDS, 30% glycerol, 0.02% bromophenol blue) and loaded on a 15% polyacrylamide gel. The gel was run in 1 × SDS running buffer (25 mM Tris, 192 mM glycine, 0.1% SDS) at 200 V for 1.5 h. Proteins were transferred onto a 0.2 μm nitrocellulose membrane (Amersham Protran, GE Healthcare, Brøndy, Denmark) in 1 × Tris-SDS transfer buffer (25 mM Tris, 192 mM glycine, 0.1% SDS) at 100 V, 4 °C for 1 h and 10 min. The membrane was blocked in 5% milk in 1 × TBST (20 mM Tris-HCl, 0.5 M NaCl, 0.0225% Tween20) for 1 h at room temperature followed by incubation with 1:400 rabbit anti-p14ARF, 1:200 mouse anti-CKII, 1:5000 human anti-TOP1, or 1:200 mouse anti-LAMIN B overnight at 4 °C. The membrane was washed 3 × 30 min in 1 × TBST followed by incubation for 1 h with 1:2000 HRP conjugated goat anti-rabbit, 1:5000 goat anti-human, or 1:1500 goat anti-mouse secondary antibody (DAKO, ThermoFisher Scientific, Roskilde, Denmark) and subsequently washed 3 × 15 min in 1 × TBST. The membrane was developed with Amersham ECL Western Blotting Detection Reagents (GE Healthcare) and Amersham Hyperfilm ECL (GE Healthcare). Quantifications were made using ImageJ (NIH, Bethesda, USA). 

### 4.8. Knockdown

AllStars negative control siRNA (#SI03650318) and siRNA targeting p14ARF (#1027416) were purchased from Qiagen. The following sequences were used:

Hs_CDKN2A 9: AAT AGT TAC GGT CGG AGG CCG
Hs_CDKN2A 12: CAC GCC CTA AGC GCA CAT TCA
Hs_CDKN2A 14: CAG AAC CAA AGC TCA AAT AAA
Hs_CDKN2A 15: TAC CGT AAA TGT CCA TTT ATA

First, 0.7 × 10^6^ Caco2 cells were seeded in complete growth medium without antibiotics. After 24 h, transfection was performed. Then, 12 μL of a 1:1:1:1 pool of 10 μM p14ARF siRNAs (Hs_CDKN2A 9, Hs_CDKN2A 12, Hs_CDKN2A 14, and Hs_CDKN2A 15) or 12 μL scrabbled siRNA (AllStars negative control siRNA) were mixed with 1 mL Opti-MEM (Gibco, ThermoFisher Scientific, Roskilde, Denmark). For each transfection 20 μL Lipofectamine RNAiMAX (ThermoFisher Scientific, Roskilde, Denmark) were mixed with 1 mL Opti-MEM. Lipofectamine and siRNA were mixed and incubated at room temperature for 15 min before being added dropwise to the cells. After 48 h, 2–4 × 10^6^ cells were re-seeded in complete growth medium and after an additional 24 h, cells were treated with 0.5 µM CPT or 0.05% DMSO as a control for 48 h before flow-cytometry analysis was performed. 

### 4.9. Quantitative PCR

RNA from sorted Caco2 cells was purified using RNeasy mini kit (Qiagen, Hilden, Germany) following the manufacturer’s instructions. The purified RNA was used for cDNA synthesis using QuantiTect Reverse Transcription Kit (Qiagen) following the manufacturer’s instructions. All qPCR reactions were performed using Platinum SYBR Green qPCR SuperMix (Invitrogen, ThermoFisher Scientific, Roskilde, Denmark) and β-actin was used as a reference gene. qPCR was performed using the following primers:

p14ARF: F: 5′-CCC GAT TGA AAG AAC CAG AGA G-3′; R: 5′-AGT TGT GGC CCT GTA GGA-3′
CKII: F: 5′-TGT CCG AGT TGC TTC CCG ATA CCT-3′; R: 5′-TGG CCA GCA TAC AAC CCA AAC TCC-3′
TOP1: F: 5′-TTC AAA GCC CAG ACG G-3′-; R: 5′-GCC ACG GAA AAG TCC A-3′
β-actin: F: 5′-GAA GGT GAA GGT CGG AGT CA-3′; R: 5′-GAG GTC AAT GAA GGG GTC AT-3′

For each specific primer pair, a qPCR master mix was prepared. Each reaction was performed in triplicate in a reaction mixture containing 7.5 µL SYBR Green, 0.03 µL ROX reference dye, 1 µL 10 μM forward and reverse primers, 1 µL H_2_O, and 5 ng cDNA. The qPCR reaction was performed on a Stratagene Mx3000P (Agilent Technologies, Glostrup, Denmark) using the following program: 15 min at 95 °C, 40 cycles of 30 s at 95 °C, 1 min at 56 °C, and 1 min at 72 °C. The run was ended with 1 min at 95 °C, 30 s at 55 °C, and 30 s at 95 °C. The amount of p14ARF, CKII, and TOP1 in CD44 positive and CD44 negative cells was calculated based on the Ct values using the following equations: ∆Ct = Ct(target) − Ct(reference) and ∆Ctexp = 2^−∆Ct^. 

### 4.10. Detection of Topoisomerase 1 Activity by Rolling-Circle Enhanced Enzyme Detection Assay

Oligonucleotides for construction of the S(hTopI) substrate and the REEAD primer were purchased from DNA Technology A/S (Aarhus, Denmark) and synthesized on a model 394 DNA synthesizer from Applied Biosystems. The sequences of the oligonucleotides are as follows:

S(hTopI): 5′-AGA AAA ATT TTT AAA AAA ACT GTG AAG ATC GCT TAT TTT TTT AAA AAT TTT TCT AAG TCT TTT AGA TCC CTC AAT GCA CAT GTT TGG CTC CGA TCT AAA AGA CTT-3′
REEAD primer: 5′-CCA ACC AAC CAA CCA AAT AAG CGA TCT TCA CAG T-3′
Detection probe: 5′-′FAM’-CCT CAA TGC ACA TGT TTG GCT CC-3′.

The REEAD assay was used to measure TOP1 activity from siRNA transfected Caco2 or siRNA transfected Caco2 CD44 negative cells, and performed essentially as previously described [50,60,61]. Briefly, CodeLink Activated HD slides were coupled with 10 µM REEAD-primer specific for the S(hTopI) substrate. TOP1 reactions were carried out in a 10 μL reaction volume containing a divalent cation depletion buffer (10 mM Tris-HCl pH 7.5, 5 mM EDTA, and 50 mM NaCl). The reaction mixtures were supplemented with 500 nM S(hTopI) DNA substrate. Reactions were initiated by the addition of whole cell extract from 10^3^ cells. The closed circles were hybridized to the slides and rolling circle amplification was performed for 1 h at 37 °C in 1 × Phi29 buffer supplemented with 10 μg/μL BSA, 10 mM dNTP, and 10 units/μL Phi29 polymerase. The reactions were stopped by washing the slides in wash buffer 1 (100 mM Tris-HCl, 150 mM NaCl, and 0.3% SDS) followed by 1 min wash in wash buffer 2 (100 mM Tris-HCl, 150 mM NaCl, and 0.05% Tween20) and dehydration in 96% EtOH. The generated rolling circle products (RCP) were detected by hybridization to 10 μM 5′-FAM-detection probe in hybridization buffer (40% formamide, 4 × SSC, 10% glycerol) for 30 minutes at 37 °C. The slides were washed in wash buffers 1 and 2, dehydrated and mounted with Vectashield without DAPI (Vector Laboratories), and visualized using a 60× objective in a fluorescence microscope (Olympus IX73). The signals detected in an average of 12 microscopic images were counted using ImageJ and the result normalized as previously reported [52]. All data were plotted as mean with SEM. 

On-Slide-REEAD assay was used to measure TOP1 activity from siRNA transfected Caco2 CD44 positive cells and performed as previously described [51]. Briefly, CodeLink Activated HD slides (Eden Prairie, Minnesota, USA) were coupled with 10 µM REEAD-primer specific for S(hTopI) and 0.5 mg/mL anti-CD44 antibody (CD44/H-CAM). Then, 2 μL of siRNA transfected Caco2 cells were added to the functionalized slides; the slides were washed by soaking in 50 mL 1 × PBS before the captured cells were lysed by adding 3 μL of lysis buffer (10 mM Tris-HCl pH 7.5, 5 mM EDTA, 0.1 mM Phenylmethylsulfonyl fluoride (PMSF) directly onto the slides followed by gentle mixing. A negative control containing only lysis buffer as substitutes for the cell lysate was included. The TOP1 mediated circularization was initiated by the addition of 1 μL of 1 µM S(hTopI). The samples were mixed by pipetting and incubated 1 h at 37 °C. Then, the rolling circle amplification and RCP detection were performed as described above for the REEAD assay of the Caco2 and Caco2 CD44 negative cells. 

## 5. Conclusions

In conclusion, our results suggest that the tumorigenic and camptothecin resistant progenitor cells of colon cancer may be targeted by combining camptothecin treatment with p14ARF inhibition. Topoisomerase I activity was slightly decreased in cells lacking the progenitor markers upon p14ARF downregulation. However, since these cells do not have tumorigenic activity the in vivo effect on chemotherapeutic response is likely to be modest. 

## Figures and Tables

**Table 1:** p14ARF knockdown increases the camptothecin sensitivity of the cancer stem-cell-like Caco2 cells.

siRNA	% Dead CD44 Positive Cells (CPT)	% Dead CD44 Negative Cells (CPT)	Total % Dead Cells (CPT)	Total % Dead Cells (DMSO)
siRNA (scramble)	25.3	74.7	19	7
siRNA (p14ARF)	38	62	45.4	32.4

Caco2 cells were transfected with siRNA (scramble) or siRNA (p14ARF) before treatment with 0.5 μM CPT or DMSO. The cells were harvested, labeled with APC conjugated anti-CD44 antibody, stained with propidium iodide (PI), and analyzed by flow-cytometry using the Beckman Cytoflex instrument. Data were analyzed and compensated using FlowJo software. After removal of cell debris and doublets by using forward scatter (FSC), side scatter (SSC), and single stain gatings, the percentages of total dead cells were estimated by the total number of PI positive cells. The percentage of CSC-like (CD44 positive) dead cells was calculated as: number of double positive stained cells (PI positive and CD44 positive)/total number of dead cells (all the PI positive cells). The percentage of non-CSC-like (CD44 negative) dead cells was calculated as: number of single positive stained cells (PI positive and CD44 negative)/total number of dead cells (all the PI positive cells).

## Supplementary Materials

The following are available online at https://www.mdpi.com/2072-6694/12/5/1240/s1, Figure S1: (**A**) Histograms showing the result of flow-cytometric analyses of Caco2 and DLD1 before or after treatment with NaBt. (I–IV) show histograms indicating the percentage of Caco2 cells that were positive (red) for either CD44 (I and III) or CD133 (II and IV) or negative (black) for both surface markers. (V–VIII) show histograms indicating the percentage of DLD1 cells that were positive (green) for either CD44 (V and VII) or CD133 (VI and VIII) or negative (black) for both surface markers. (**B**) Table showing the percentage of cells characterized by the CD44 or CD133 markers in Caco2 and DLD1 as estimated by flow-cytometry in the presence or absence of NaBt treatment as indicated. (**C**) Graphical depiction of flow-cytometry analysis of the DLD1 cells. DLD1 cells were sorted in the two subpopulations of CD133 negative and CD133 positive cells and cultivated for the indicated time periods, up to 60 days, before they were stained with PE conjugated anti-CD133 antibody and analyzed by flow-cytometry. The results are plotted as percentage of CD133 positive cells as a function of cultivation time. Black circles show the result of the FACS sorted CD133 negative cells; green squares show the results of FACS sorted CD133 positive cells; blue triangles show the results obtained by using unsorted DLD1; green arrow shows the percentage of CD133 positive cells (CD133 pos_start_) present in the CD133 positive FACS sorted cells at the day of the FACS sorting (=time 0); blue arrows show the percentage of CD133 positive cells present in the unsorted DLD1 cells (Unsorted_start_) at the day of the FACS sorting (=time 0); black arrow shows the percentage of CD133 positive cells (CD133 neg_start_) present in the CD133 negative FACS sorted cells at the day of the FACS sorting (=time 0). (**D**) Flow-cytometry analysis of the Caco2 cells. Caco2 cells were FACS sorted in the two subpopulations of CD44 negative and CD44 positive. The sorted cell subpopulations were cultivated for the indicated time intervals, stained with PE conjugated anti-CD44 antibody, and analyzed by flow-cytometry. The results are depicted as percentage CD44 positive cells as a function of cultivation time. Black circles show the results of FACS sorted Caco2 CD44 negative cells; red squares show the results obtained with FACS sorted Caco2 CD44 positive cells; blue triangles show the results obtained with unsorted Caco2 cells; red arrow shows the percentage of CD44 positive cells (CD44 pos_start_) present in the CD44 positive FACS sorted cell population at the day of the FACS sorting (=time 0); blue arrows show the percentage of CD44 positive cells present in the unsorted Caco2 cells (Unsorted_start_) at the day of the the FACS sorting (=time 0); black arrow shows the percentage of CD44 positive cells (CD44 neg_start_) present in the CD44 negative FACS sorted cells at the day of the FACS sorting (=time 0), Figure S2: CPT sensitivity and TOP1 activities in DLD1 cell subpopulations. (**A**) Survival assay of the FACS sorted CD133 negative or CD133 positive cells. The cells were incubated with DMSO (the solvent of CPT) or 0.2, 0.4, 0.8, 1.6, or 3.2 μM of CPT for 72 h before the percentage of viable cells was measured by the PrestoBlue method. Results were normalized to the DMSO-treated sample for each cell subpopulation and plotted as mean +/− SEM. The differences were all significant with *p* < 0.0001 for each bar (Welch’s *t*-test). (**B**) Graphical depiction of the REEAD assay. The S(hTopI) substrate folds into a dumbbell shaped structure with a preferential TOP1 cleavage site indicated by the green arrow (upper left). Upon TOP1 mediated cleavage and religation, the s(hTopI) is converted to a closed circle that can be amplified by rolling circle amplification mediated by the Phi29 polymerase (black dot, right). The resulting rolling circle amplification products are visualized by incorporation of radioactively labeled α^32^P-dATP. (**C**) Measurement of TOP1 activity by REEAD in the nuclear extracts from CD133 negative (black bars) and CD133 positive (grey bars) FACS sorted cell subpopulations, respectively. The graph shows the quantification of the number of REEAD signals (counted by using the Image software) relative to the total amount of proteins. The graph is the average of three independent experiments with the results plotted as mean +/− SEM. * *p* < 0.016, (Welch’s *t*-test), Figure S3: Analysis of DLD1 expressed TOP1 PTM pattern. (**A**) Comparative 1D Western blot analysis of the TOP1 expression level in extracts from unsorted or FACS sorted DLD1 cells. Equal amount of protein loading was ensured by the blot development with anti-tubulin antibodies. (**B**) The TOP1 PTM patterns in the cellular extract of Caco2 cells were revealed by 2D WB. The TOP1 PTM patterns and the effect of dephosphorylation by λPpase treatment of the TOP1 in extracts from unsorted DLD1 cells (**C**) or FACS sorted CD133 negative (**D**) or CD133 positive (**E**) DLD1 cells were assessed by 2D WB. All blots were developed with anti-TOP1 antibodies. Equal amounts of cellular extract from the CD133 negative (**D**) or CD133 positive (**E**) fractions were loaded based on the analysis performed in (A). The presence or absence of phosphatase treatment for each sample is indicated by “λPpase^+^” and “λPpase^−^”. The black arrows indicate the position of TOP1 isoforms that are sensitive towards λPpase treatment. The direction of protein separation (pI 3 to 10) is specified at the top of the images, Figure S4: CKII activity measurement in extracts from Caco2 and DLD1 cells. (**A**) Gel electrophoresis of the products obtained after incubation of Caco2 nuclear cell extracts with p25 (TOP1 a.a. 1–206) in the presence of γ ^32^P-ATP over time. The bands represent the intensity of the radiolabeled p25 after being phosphorylated by cell extracts from cells treated with DMSO (left) or with 10 μM TBB (right) in the presence of a broad range of kinase inhibitors specific for other kinases than CKII. The lower panel is a graphical depiction of the results. Radiolabeled bands from 3 individual gels were quantified by densitometry using QuantityOne software and the results plotted as mean +/− SEM. Black line: DMSO; grey line: TBB. (**B**) same as (A) except that extracts from DLD1 cells were analyzed, Figure S5: Analysis of TOP1 in Caco2 cells with or without TBB treatment. (**A**) and (**B**) the effect of dephosphorylation by λPpase on the TOP1 PTMs pattern was assessed by 2D Western blot. The blots were developed with anti-TOP1 antibody. Equal amounts of cellular extracts from the CD44 negative (**A**) or CD44 positive (**B**) fractions were loaded based on the analysis performed in (**C**). Subpopulations from cells propagated in the presence of DMSO (marked DMSO) or TBB (marked TBB) were analyzed before (λPpase^-^) or after (λPpase^+^) treatment. The black arrows indicate the position of TOP1 isoforms that are sensitive towards λPpase treatment. The red arrows indicate the position of TOP1 isoforms that are not sensitive towards λPpase treatment. The direction of protein separation (pI 3 to 10) is specified at the top of the images. (**C**) A 1D Western blot analysis of cellular extracts from unsorted or FACS sorted Caco2 cells treated with DMSO or TBB. The loading of equal amounts of protein was ensured by blot development with anti-tubulin antibodies. (**D**) Activity of TOP1 measured in extracts from unsorted Caco2 cells, with and without TBB treatment. S(hTopI) was incubated with nuclear cell extract from 10^6^ cells and the activity measured using the REEAD assay [12]. REEAD signals were counted using ImageJ software and the results were plotted as mean +/− SEM. **** *p* < 0.0001 (Welch’s *t*-test), Figure S6: (**A**) Left panel, Western blot analysis of whole cell extracts from non-CSC-like (CD44 negative) or CSC-like (CD44 positive) cells, developed by using anti-TOP1, anti-LAMIN B (as a loading control), or anti-p14ARF antibodies. Right panel, graphical depiction showing the results of the densitometric quantification of the bands shown in the Western blot. The intensity of the TOP1 and p14ARF specific bands were normalized relative to the intensity of the LAMIN B bands. (**B**) Graphical depiction of the results for qRT-PCR analysis of p14ARF mRNA extracted from non-CSC-like (CD44 negative) or CSC-like (CD44 positive) cells. p14ARF gene expression was calculated based on the Ct values using the following equations: ∆Ct = Ct(target) − Ct(reference) and ∆Ct^exp^ = 2^−∆Ct^. The results were plotted as fold change and represent the mean +/− SEM of three independent experiment using Welch’s *t*-test, Figure S7: TOP1 activity with or without downregulation of p14ARF in the Caco2, non-CSC-like, and CSC-like cells. Left graph, measurement of TOP1 activity in the whole cell extracts from Caco2 or Caco2 non-CSC-like (CD44 negative) cells transfected with siRNA (scramble) (black bars) or siRNA (p14ARF) (grey bars). The CD44 negative cells were obtained by NaBt treatment giving approx. 100% cell differentiation (Figure 1A). TOP1 activity was measured by the REAAD assay, and the REEAD signal was quantified by ImageJ software and plotted as mean +/− SEM. Right graph, measurement of TOP1 activity in the whole cell extracts from Caco2 CSC-like (CD44 positive) cells transfected with siRNA (scramble) (black bars) or siRNA (p14ARF) (grey bars). The CD44 positive cells were captured onto a glass slide by using anti-CD44 antibody. Following cell lysis, the TOP1 activity was measured by using the On-Slide-REEAD assay as described previously [51]. The REEAD signals were quantified by ImageJ software and plotted as mean +/− SEM. In both experiments the number of signals counted were normalized to the number of signals obtained when analyzing a standard sample containing circularized substrate obtained by incubation with purified TOP1. Hence, the results are shown as TOP1 activity relative to the standard. This was done to avoid experimental noise resulting from slide-to-slide variations as previously described [31,50,52], Figure S8: Effect of the p14ARF downregulation on the non-CSC-like and CSC-like CPT sensitivity. Caco2 cells were transfected with siRNA (scramble) (upper panels) or siRNA (p14ARF) (lower panels) and treated with DMSO (left panels) or 0.5 μM CPT. Cells were harvested and labeled with APC conjugated anti-CD44 antibody and stained with propidium iodide (PI) before they were analyzed by flow-cytometry using the Beckman Cytoflex instrument. Data were analyzed using FlowJo software and compensated by using single stained samples. The dot plots show compensated PI values (Comp-FL2-A:PI, x-axis) versus compensated CD44-APC values (Comp-FL3:CD44 APC, y-axis). In each panel the total number of analyzed cells is reported at the bottom of the dot plots, Figure S9: p14ARF knockdown prevents accumulation of CSC-like Caco2 cells upon CPT treatment. The Caco2 cells were transfected with siRNA (scramble) (top panels) or siRNA (p14ARF) (bottom panels) and treated with DMSO (left panels) or 0.5 μM CPT (right panels) for 72 h before they were stained with PE conjugated anti-CD44 antibody. The cells were analyzed by flow-cytometry using the Beckman Cytoflex instrument. Cell debris and doublet were removed from the analysis using FSC and SSC gatings. The percentages of CSC-like Caco2 cells (CD44 positive) are shown in red, Figure S10: Original blots. (**A**) Original blot of the Western blot shown in Figure 3A. (**B**) Original blot of the Western blot shown in Figure 4A. (**C**) Original blot of the Western blot shown in Figure S6A. For all blots molecular weights are indicated at the right of the blots.

## Abbreviations

CPT	camptothecin
TOP1	human topoisomerase I
CSC	cancer stem cell
NaBt	sodium butyrate
TBB	4,5,6,7-tetrabromobenzotriazole
p14ARF	ARF tumor suppressor
CKII	Casein Kinase II
APC	allophycocyanin
FACS	Fluorescence Activated Cell Sorting
FSC	Forward scatter
SSC	Side scatter
PMSF	Phenylmethylsulfonyl fluoride
GST	Glutathione S-transferase
