# Supplementary material for: Different Camptothecin Sensitivities in Subpopulations of Colon Cancer Cells Correlate with Expression of Different Phospho-Isoforms of Topoisomerase I with Different Activities
Source: Cancers (Basel). 2020 May 14;12(5):1240. doi: 10.3390/cancers12051240 (PMC7281652; doi:10.3390/cancers12051240)
Supplement: Supplementary file 1 [file cancers-12-01240-s001.pdf]

cancers

MDPI

Different Camptothecin Sensitivities in Subpopulations of Colon Cancer Cells Correlate with Expression of Different Phospho-Isoforms of Topoisomerase I with Different Activities

Cinzia Tesauro [1,†], Josephine Geertsen Keller [1,2,†], Irina Gromova [3], Pavel Gromov [3], Rikke Frøhlich [1], Jens Uldum Erlandsen [1], Anni H. Andersen [1], Magnus Stougaard [2,4] and Birgitta R. Knudsen [1,*]

[1] Department of Molecular Biology and Genetics, C. F. Møllers Allé 3, Bldg. 1131, Aarhus University, 8000 Aarhus C, Denmark; ctesauro@mbg.au.dk (C.T.); jgk@clin.au.dk (J.K.); psg@cancer.dk (R.F.); jens@uldum-erlandsen.dk (J.U.E.); aha@mbg.au.dk (A.H.A.)

[2] Department of Clinical Medicine, Aarhus University, Aarhus, 8000 Denmark; magnstou@rm.dk

[3] Genome Integrity Unit, Danish Cancer Society Research Center, Breast Cancer Group, Strandboulevarden 49 DK-2100 Copenhagen, Denmark; iig@cancer.dk (I.G.); psg@cancer.dk (P.G.);

[4] Department of Pathology, Aarhus University Hospital, Aarhus, 8000, Denmark

† These authors contributed equally

* Correspondence: brk@mbg.au.dk

Supplementary S1

Caco2 and DLD1 can be divided into subpopulations based on the expression of cell surface markers.

Cell subpopulations of Caco2 and DLD1 characterized by different expression of the cell surface markers CD44 and CD133, which are well described markers of stemness in colon cancer derived cell lines, were characterized with respect to NaBt induced differentiation and capability of asymmetric division.

For the analyses of NaBt sensitivity, the cell lines were stained with PE-conjugated anti CD44 or CD133 antibodies. The relative numbers of CD44 positive or CD133 positive cells were determined by flow-cytometry before or after treatment with NaBt. As evident from Supplementary Figure S1A and B untreated Caco2 contained approximately 23% CD44 positive and 97% CD133 positive cells, while DLD1 contained 60% CD44 positive and 6% CD133 positive cells. Upon NaBt treatment of Caco2, the fraction of CD44 positive cells were reduced to 0% and the fraction of CD133 positive cells to 42%. This is in agreement with previous observations [1–3] that suggested that CD44 is the best single marker of stemness in Caco2. In DLD1, the fraction of CD133 positive cells was reduced from 6% to 0% by treatment with NaBt, while the fraction of CD44 positive cells was reduced from 60% to 54% (Supplementary Figure S1 and B). We therefore decided to isolate the subpopulations of DLD1 based on CD133 expression. This is consistent with other reports indicating that CD133 is a single marker of stemness for the DLD1 cell line [4,5].

To investigate if asymmetric cell division characterized the CD133 positive DLD1 cells and the CD44 positive Caco2 cells, a DLD1 and a Caco2 cell culture was FACS sorted based on surface marker expression. This was to generate pure CD133 positive or CD133 negative DLD1 starting populations (termed $CD133pos_{start}$ or $CD133neg_{start}$) and pure CD44 positive or CD44 negative Caco2 starting populations (termed $CD44pos_{start}$ or $CD44neg_{start}$). The purity of the obtained cell subpopulations was determined by flow-cytometry after FACS sorting and the results depicted at time 0 in the graphs shown in Supplementary Figures S1C and D. The sorted cell subpopulations were further cultured for 60 days alongside an unsorted culture of each cell line. To follow the progression of the different cultures, cells were taken out at the indicated time intervals post separation and analyzed for the expression of the relevant surface markers. As evident from Supplementary Figure S1C, the distribution of CD133 positive DLD1 cells in the $CD133pos_{start}$ population decreased over time to 6% corresponding to the stable proportion of CD133 positive cells in DLD1. This result is consistent with the ability of CD133 positive cells to divide asymmetrically and produce CD133 negative cells. In

CD133neg $_{start}$, the proportion of CD133 positive cells fluctuated around 0% in the duration of the experiment supporting the inability of this cell subpopulation to divide asymmetrically. The progression of CD44pos$_{start}$ in Caco2 was reminiscent to that of CD133pos$_{start}$ in DLD1 with the proportion of CD44 positive cells decreasing over time and stabilizing at approximately 20%, which corresponded to the stable proportion of CD44 positive cells in Caco2. Also, the progression of CD44neg$_{start}$ was like the progression of CD133neg$_{start}$ (compare Supplementary Figure S1C with S1D), although the results were not as clear as for CD133neg$_{start}$. This is due to the tendency of Caco2 cells to adhere in clumps making it difficult to avoid small contaminations of CD44 positive cells in the sorted CD44 negative cell fraction. Note that CD44neg$_{start}$ contained approx. 7% CD44 positive cells.

With regard to sensitivity towards NaBt treatment and the asymmetric cell division, the CD133 positive DLD1 and the CD44 positive Caco2 cell subpopulations behaved similarly and both demonstrated the characteristics previously reported for CSC-like cells in cancer derived cells lines [6]. This is also in agreement with the tumor initiating capacity of these cell subpopulations as reported elsewhere [4,7,8].

The evolution of the FACS sorted CD133 positive DLD1 and the CD44 positive Caco2 cell subpopulations is in accordance with studies by Gupta *et al.* [6]. They showed that CSC-like subpopulations of breast cancer derived cell lines return to the equilibrium phenotypic proportions over time according to the Markov model [6]. Based on this and other studies, it is well accepted that besides tumor initiating capacity, CSC can be defined by the ability to divide asymmetrically and give rise to other CSC as well as more differentiated non-CSC [9–11]. Hence, although the selection of the DLD1 subpopulations on the basis of CD133 expression and the Caco2 cell subpopulations on the basis of CD44 expression at first glance may seem very different, this difference is illusive. Indeed, all Caco2 cells, including the selected CD44 positive cells, were also CD133 positive. Based on this, the observed behavior of the cell subpopulations described above, and the available literature [3,8], we argue that the cell subpopulations with CSC-like characteristics may best be selected on the basis of CD44 expression in Caco2 and CD133 expression in DLD1 cells.

A

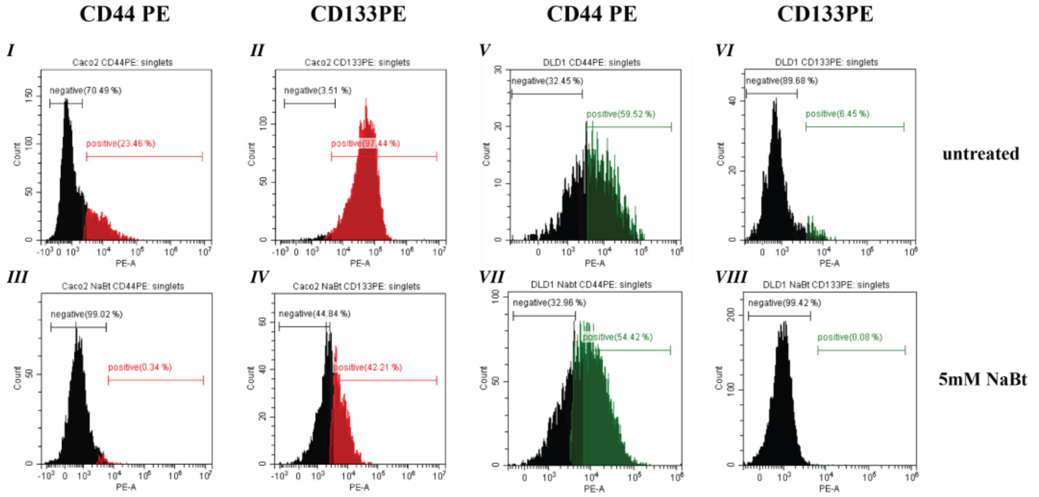

B

	Caco2				DLD1			
	CD133		CD44		CD133		CD44	
	-	+	-	+	-	+	-	+
-NaBt	4%	97%	70%	23%	90%	6%	32%	60%
+NaBt	45%	42%	99%	0%	99%	0%	33%	54%

C D

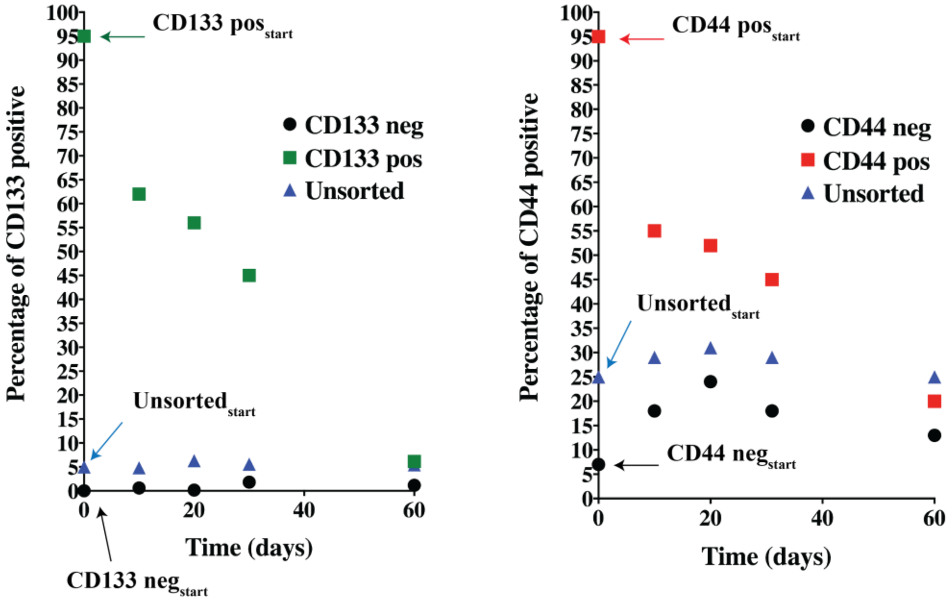

Figure S1. (**A**) Histograms showing the result of flow-cytometric analyses of Caco2 and DLD1 before or after treatment with NaBt. (I–IV) show histograms indicating the percentage of Caco2 cells that were positive (red) for either CD44 (I and III) or CD133 (II and IV) or negative (black) for both surface markers. (V–VIII) show histograms indicating the percentage of DLD1 cells that were positive (green) for either CD44 (V and VII) or CD133 (VI and VIII) or negative (black) for both surface markers. (**B**) Table showing the percentage of cells characterized by the CD44 or CD133 markers in Caco2 and DLD1 as estimated by flow-cytometry in the presence or absence of NaBt treatment as indicated (**C**) Graphical depiction of flow-cytometry analysis of the DLD1 cells. DLD1 cells were sorted in the two subpopulation CD133 negative and CD133 positive cells and cultivated for the indicated time periods, up to 60 days, before they were stained with PE conjugated anti CD133 antibody and analyzed by

flow-cytometry. The results are plotted as percentage of CD133 positive cells as a function of cultivation time. Black circles, show the result of the FACS sorted CD133 negative cells; Green squares, show the results of FACS sorted CD133 positive cells; Blue triangles, show the results obtained by using unsorted DLD1. Green arrow: percentage of CD133 positive cells (CD133 pos_{start}) present in the CD133 positive FACS sorted cells at the day of the FACS sorting (=time 0); Blue arrow: Percentage of CD133 positive cells present in the unsorted DLD1 cells ($Unsorted_{start}$) at the day of the FACS sorting (=time 0); Black arrow: percentage of CD133 positive cells (CD133 neg_{start}) present in the CD133 negative FACS sorted cells at the day of the FACS sorting (=time 0). (**D**) Flow-cytometry analysis of the Caco2 cells. Caco2 cells were FACS sorted in the two subpopulations CD44 negative and CD44 positive. The sorted cell subpopulations were cultivated for the indicated time intervals, stained with PE conjugated anti CD44 antibody and analyzed by flow-cytometry. The results are depicted as percentage CD44 positive cells as a function of cultivation time. Black circles, show the results of FACS sorted Caco2 CD44 negative cells; Red squares, show the results obtained with FACS sorted Caco2 CD44 positive cells; Blue triangles, show the results obtained with unsorted Caco2 cells. Red arrow: percentage of CD44 positive cells (CD44 pos_{start}) present in the CD44 positive FACS sorted cell population at the day of the FACS sorting (=time 0); Blue arrow: Percentage of CD44 positive cells present in the unsorted Caco2 cells ($Unsorted_{start}$) at the day of the the FACS sorting (=time 0); Black arrow: percentage of CD44 positive cells (CD44 neg_{start}) present in the CD44 negative FACS sorted cells at the day of the FACS sorting (=time 0).

Supplementary S2

The CD133 Positive DLD1 Cells are Camptothecin Resistant and Possess Low Intracellular Topoisomerase 1 Activity.

To investigate if the two subpopulations of DLD1 cells responded differently to CPT treatment (as it was observed in Caco2), a DLD1 cell culture was stained with PE conjugated anti CD133 antibody and FACS sorted on the basis of CD133 expression. The purity of the obtained cell populations was confirmed by re-analysis after sorting using flow-cytometry to approx. 97% and 99% for the CD133 positive and CD133 negative cell populations, respectively (data not shown).

The CPT sensitivity of the two DLD1 cell subpopulations was measured in a standard survival assay after 72 hours of cultivation in the presence of 0 to 3.2 μM CPT and the result depicted as a bar chart in Supplementary Figure S2A. As evident from the figure, the CD133 positive cells were considerably less sensitive towards CPT treatment than the CD133 negative cells (compare the grey with the black bars). This is in agreement with the results obtained for the CD44 positive Caco2 cells (see Figure 1 and [1].

The activity of TOP1 in extracts from the FACS sorted CD133 positive and CD133 negative DLD1 cells was measured using the REEAD assay as previously described [1,12] and shown in Supplementary Figure S2B.

Supplementary Figure S2C shows a graphical depiction of the results obtained when measuring the TOP1 activity in extracts corresponding to 15 x 10^3, 7.5 x 10^3 or 3.75 x 10^3 of the FACS sorted CD133 positive or CD133 negative cells using the REEAD assay. As evident from the figure, extract from CD133 positive cells contained approximately 30% less TOP1 activity than extract from the same number of CD133 negative cells.

Taken together, the results shown in Supplementary Figure S2 demonstrate that the decreased CPT sensitivity of CD133 positive relative to CD133 negative DLD1 cells correlated with a relatively low enzyme activity of TOP1 in these cells. This is in agreement with the observations made in the CD44 defined subpopulations of Caco2 [1].

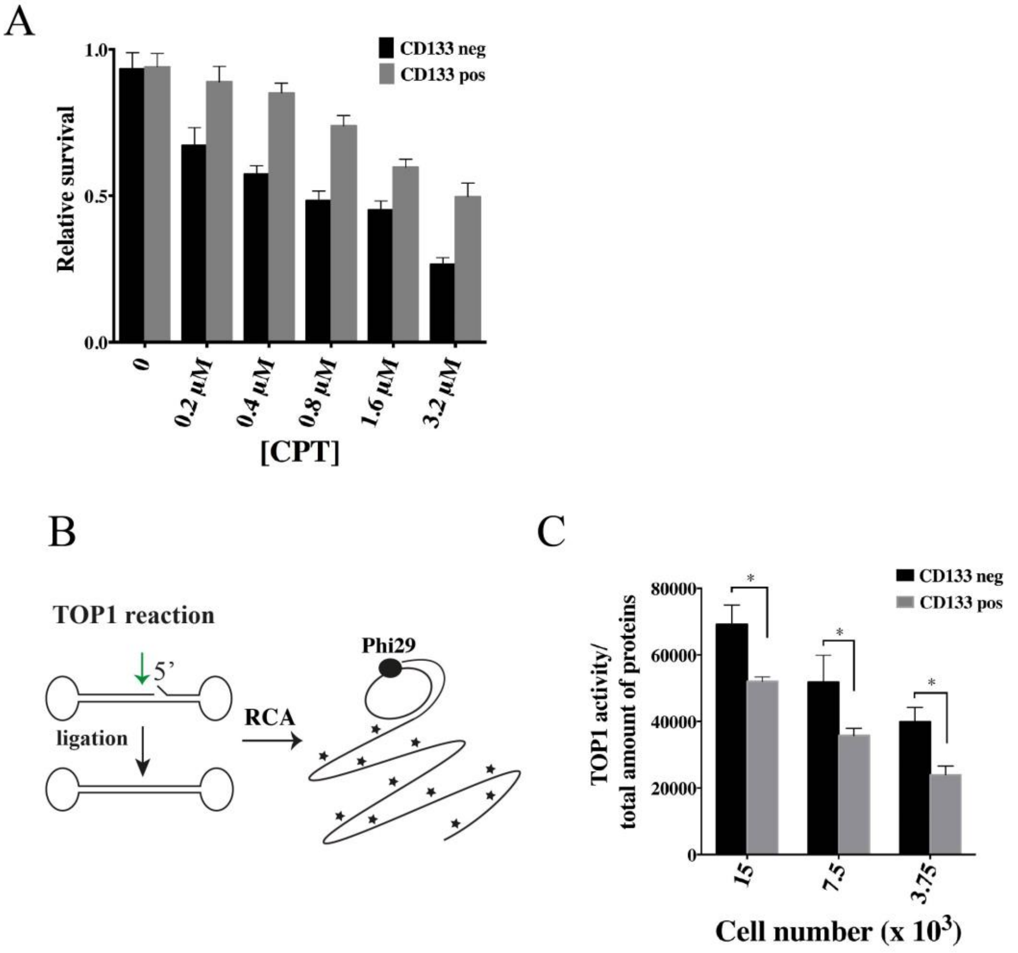

Figure S2. CPT sensitivity and TOP1 activities in DLD1 cell subpopulations. **A)** Survival assay of the FACS sorted CD133 negative or CD133 positive cells. The cells were incubated with DMSO (the solvent of CPT) or 0.2, 0.4, 0.8, 1.6 or 3.2 μM of CPT for 72 hours before the percentage of viable cells was measured by the PrestoBlue method. Results were normalized to the DMSO treated sample for each cell subpopulation and plotted as mean +/- SEM. The differences are all significant with $P<0.0001$ for each bar (Welch's t-test) **B)** Graphical depiction of the REEAD assay. The S(hTopI) substrate folds into a dumbbell shaped structure with a preferential TOP1 cleavage site indicated by the green arrow (upper left). Upon TOP1 mediated cleavage and re-ligation, the s(hTopI) is converted to a closed circle that can be amplified by rolling circle amplification mediated by the Phi29 polymerase (black dot, right). The resulting rolling circle amplification products are visualized by incorporation of radioactively labeled α^{32}P-dATP. **C)** Measurement of TOP1 activity by REEAD in the nuclear extracts from CD133 negative (black bars) and CD133 positive (grey bars) FACS sorted cell subpopulations, respectively. The graph shows the quantification of the number of REEAD signals (counted by using the Image software) relative to the total amount of proteins. The graph is the average of three independent experiments with the results plotted as mean +/- SEM. *$P<0.016$, (Welch's t-test).

Supplementary S3

The Phosphorylation Profile of Topoisomerase 1 Differed in the two DLD1 Cell Subpopulations.

The phosphorylation profiles of TOP1 in the unsorted DLD1 cells as well as in FACS sorted CD133 negative or CD133 positive cells were investigated. The total amount of TOP1 in whole cell extracts from DLD1 was measured by 1D Western blotting (Supplementary Figure S3A). The results showed that CD133 positive cells expressed less TOP1 as compared to the CD133 negative counterpart (compare lane 3 with 2). This might at least in part explain the differences between the two subpopulations regarding the CPT cell sensitivity and TOP1 activity (see Supplementary Figure

S2). However, post translational modifications (PTMs) may also affect TOP1 activity as well as TOP1 CPT susceptibility, which are both expected to affect the cellular CPT sensitivity.

A 2D Western blot was performed on extracts from unsorted Caco2 cells and unsorted or FACS sorted DLD1 cells in order to compare the TOP1 PTM patterns. In general, the revealed TOP1 PTM patterns in extract from unsorted DLD1 cells was less complex as compared to the TOP1 PTM patterns in extract from Caco2 cells (compare Supplementary Figure S3B and C, upper panel).

The CD133 positive cells displayed a pattern of TOP1 isoforms that resembles the pattern of unsorted DLD1 cells (compare upper panel of Supplementary Figure S3E with C), whereas the TOP1 spot distribution of CD133 negative cells was more complex and, hence, more similar to that observed for Caco2 cells (compare upper panel of Supplementary Figure S3D with B).

As seen when comparing the upper and lower panels of Supplementary Figure S3C, D and E, the λPpase treatment removed several of the most acidic spots (marked with black arrows) of TOP1 in extracts from unsorted as well as from both FACS sorted DLD1 cell fractions indicating that these spots corresponded to phosphorylated forms of TOP1.

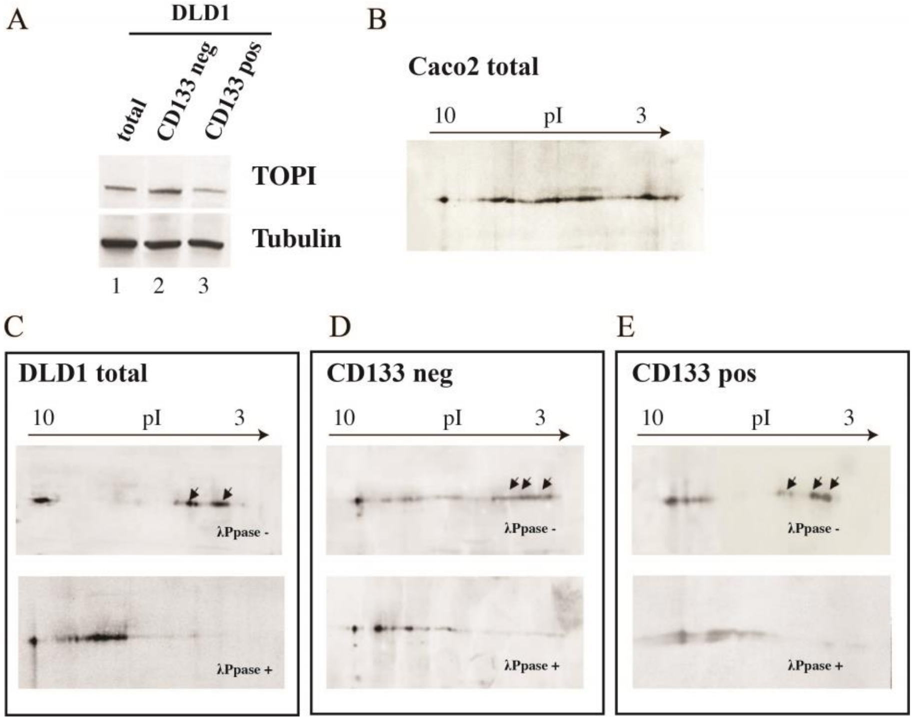

Figure S3. Analysis of DLD1 expressed TOP1 PTM pattern. **A)** Comparative 1D Western blot analysis of the TOP1 expression level in extracts from unsorted or FACS sorted DLD1 cells. Equal amount of protein loading was ensured by the blot development with anti-tubulin antibodies. **B)** The TOP1 PTM patterns in the cellular extract of Caco2 cells was revealed by 2D WB. The TOP1 PTM patterns and the effect of de-phosphorylation by λPpase treatment of the TOP1 in extracts from unsorted DLD1 cells **(C)** or FACS sorted CD133 negative **(D)** or CD133 positive **(E)** DLD1 cells was assessed by 2D WB. All blots were developed with anti TOP1 antibodies**.** Equal amount of cellular extracts from the CD133 negative **(D)** or CD133 positive **(E)** fractions was loaded based on the analysis performed in **(A).** The presence or absence of phosphatase treatment for each sample is indicated by "λPpase^{+}" and " λPpase^{-}". The black arrows indicate the position of TOP1 isoforms that are sensitive towards λPpase treatment. The direction of protein separation (pI 3 to 10) is specified at the top of the images.

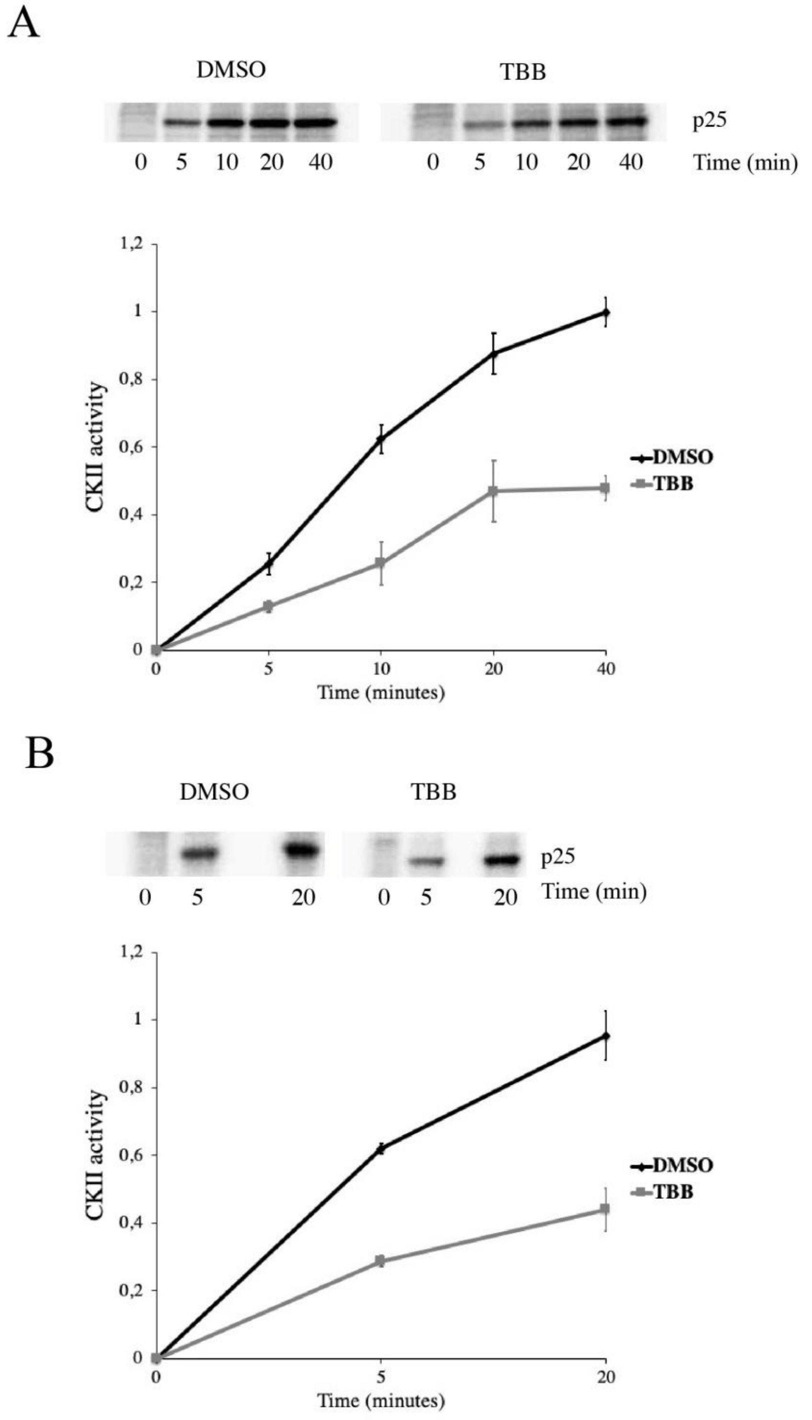

Figure S4. CKII activity measurement in extracts from Caco2 and DLD1 cells **A)** Gel electrophoresis of the products obtained after incubation of Caco2 nuclear cell extracts with p25 (TOP1 a.a. 1-206) in the presence of γ ^{32}P-ATP over time. The bands represent the intensity of the radiolabelled p25 after being phosphorylated by cells extracts from cell treated with DMSO (left) or with 10 μM TBB (right) in the presence of a broad range of kinase inhibitors specific for other kinases than CKII. Lower panel, is a graphical depiction of the results. Radiolabelled bands from 3 individual gels were quantified by densitometry using QuantityOne software and the results plotted as mean +/- SEM. Black line: DMSO; grey line: TBB. **B)** same as A except that extracts from DLD1 cells were analyzed.

Supplementary S5

TBB Treatment Affected the Phosphorylation State and the Activity of Topoisomerase 1 in Caco2 Cell Subpopulations.

To determine whether the differential CPT responses of the TBB treated or untreated cell populations could be related to the phosphorylation profile of the TOP1 protein, extracts from the differently treated cells were analyzed by 2D gel electrophoresis followed by 2D Western blot with anti TOP1 antibodies (Figure S5A and B). Also, we confirmed that the total amount of TOP1 was comparable in the extracts from unsorted or sorted CD44 positive or CD44 negative cell subpopulations both before and after TBB treatment (see Supplementary Figure S5C).

2D Western blot of proteins extracted from either CD44 negative or CD44 positive cell sub-populations revealed a complex TOP1 PTM pattern displayed as numerous isoelectric spots (Supplementary Figure S5A and B). In agreement with our previous report [1], the 2D Western blot showed a more complex TOP1 isoform pattern in the CD44 positive cells as compared to the CD44 negative cell counterparts (see the upper left images in Supplementary Figure S5A and B). Dephosphorylation experiments showed that some of the spots, corresponding to TOP1 phosphorylated forms (indicated by black arrows), vanished as a result of the λPpase treatment (Supplementary Figure S5A and B, compare the upper and the lower panels). The other group of spots (indicated by red arrows) did not change their positions after the de-phosphorylation reaction, indicating that these TOP1 isoforms resulted from other types of PTMs (Supplementary Figure S5A and B, compare the spots in the upper and the lower panels). As seen in Figure S5A and B this unknown type of PTMs was notably higher in TBB treated cells. Whether this is due to inhibition of the CKII or is a result of other effects of TBB treatment remains unknown.

The spots corresponding to phosphorylated TOP1 isoforms (λPpase sensitive) in cells treated with TBB exhibited distinctly lower intensities as compared to untreated cells (see Supplementary Figure S5, compare the upper left and right panels in A and B). This implies a lower phosphorylation status of TOP1 in TBB treated cells due to inhibition of CKII activity. These results are in line with the reduced CKII activity observed in the TBB treated cells (Supplementary Figure S4).

In accordance with previous *in vitro* analyses [1], the reduced phosphorylation state of TOP1 upon TBB treatment reduced the activity of TOP1 in extracts from treated cells as measured by REEAD (Supplementary Figure S5D).

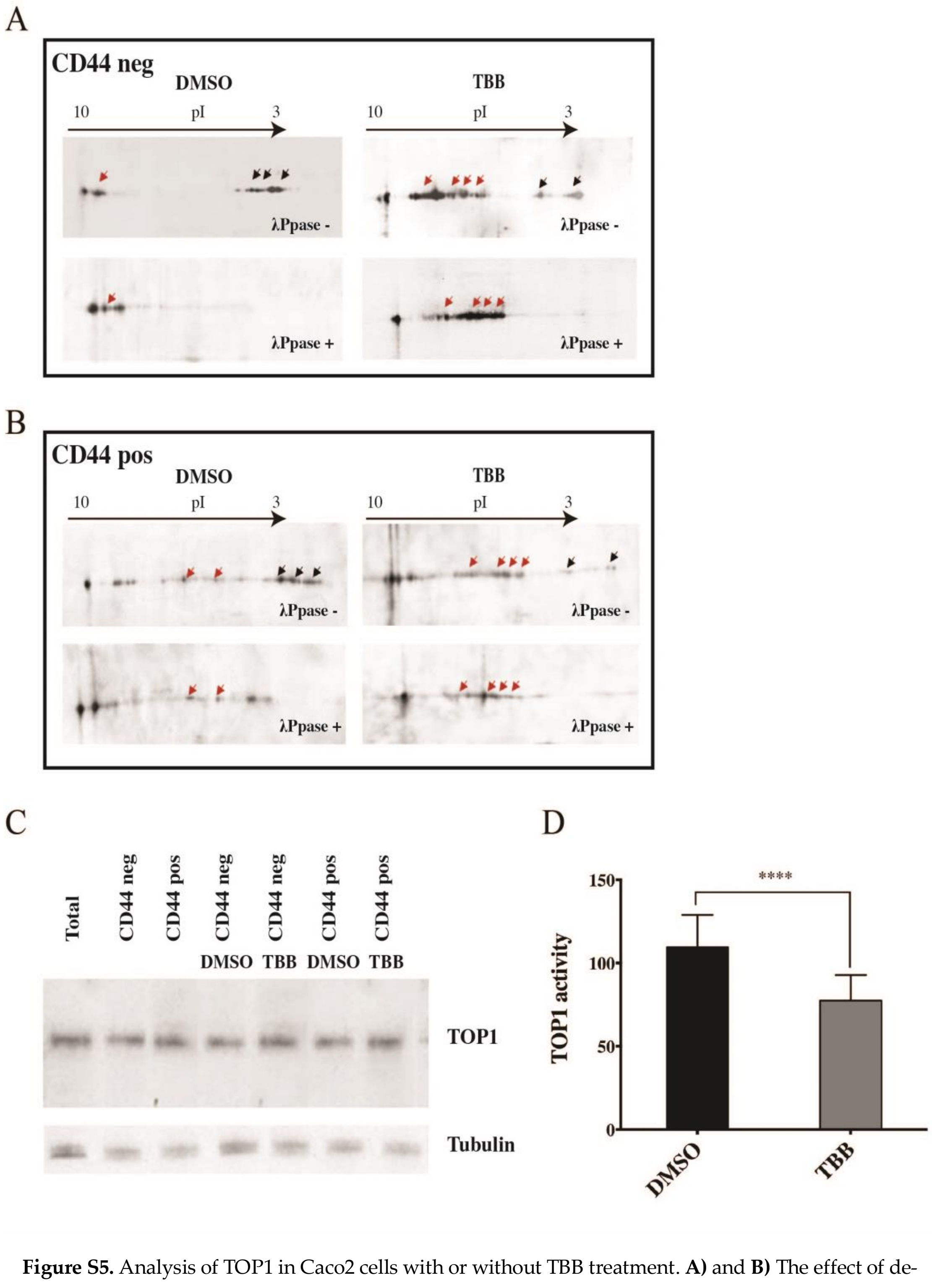

Figure S5. Analysis of TOP1 in Caco2 cells with or without TBB treatment. **A)** and **B)** The effect of dephosphorylation by λPpase on the TOP1 PTMs pattern was assessed by 2D Western blot. The blots were developed with anti TOP1 antibody. Equal amounts of cellular extracts from the CD44 negative **(A)** or CD44 positive **(B)** fractions were loaded based on the analysis performed in **C**. Subpopulations from cells propagated in the presence of DMSO (marked DMSO) or TBB (marked TBB) were analyzed before (λPpase⁻) or after (λPpase⁺) treatment. The black arrows indicate the position of TOP1 isoforms that are sensitive towards λPpase treatment. The red arrows indicate the position of TOP1 isoforms that are not sensitive towards λPpase treatment. The direction of protein separation (pI 3 to 10) is specified at the top of the images. **C)** An 1D Western blot analysis of cellular extracts from unsorted or FACS sorted Caco2 cells treated with DMSO or TBB. The loading of equal amount of protein was ensured by blot development with anti-tubulin antibodies. **D)** Activity of TOP1 measured in extracts

from unsorted Caco2 cells, with and without TBB treatment. S(hTopI) was incubated with nuclear cell extract from 10^6 cells and the activity measured using the REEAD assay [12]. REEAD signals were counted using ImageJ software and the results were plotted as mean +/- SEM. ****P<0.0001 (Welch's t-test).

Supplementary S6

Expression level of p14ARF in Caco2 cell subpopulations

The expression level of p14ARF in the CSC and non-CSC-like cell subpopulation of Caco2 was measured at the mRNA level using qRT-PCR and at the protein level using WB with an anti p14ARF antibody. As evident from Supplementary Figure S6 the two subpopulations of Caco2 expressed equal amounts of p14ARF.

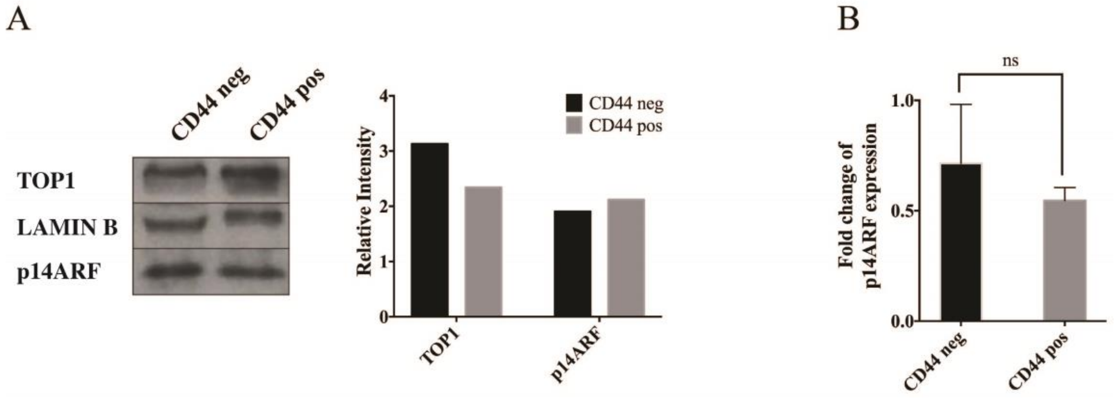

Figure S6. A) Left panel, Western blot analysis of whole cell extracts from non-CSC-like (CD44 neg) or CSC-like (CD44 pos) cells, developed by using anti TOP1, anti LAMIN B (as a loading control) or anti p14ARF antibodies. Right panel, graphical depiction showing the results of the densitometric quantification of the bands shown in the Western blot. The intensity of the TOP1 and p14ARF specific bands were normalized relative to the intensity of the LAMIN B bands. **B)** Graphical depiction of the results for qRT-PCR analysis of p14ARF mRNA extracted from non-CSC-like (CD44 neg) or CSC-like (CD44 pos) cells. p14ARF gene expression was calculated based on the Ct values using the following equations: $\Delta Ct = Ct(target) - Ct(reference)$ and $\Delta Ct^{exp} = 2^{-\Delta Ct}$. The results were plotted as fold change and represent the mean +/- SEM of three independent experiment using Welch's t-test.

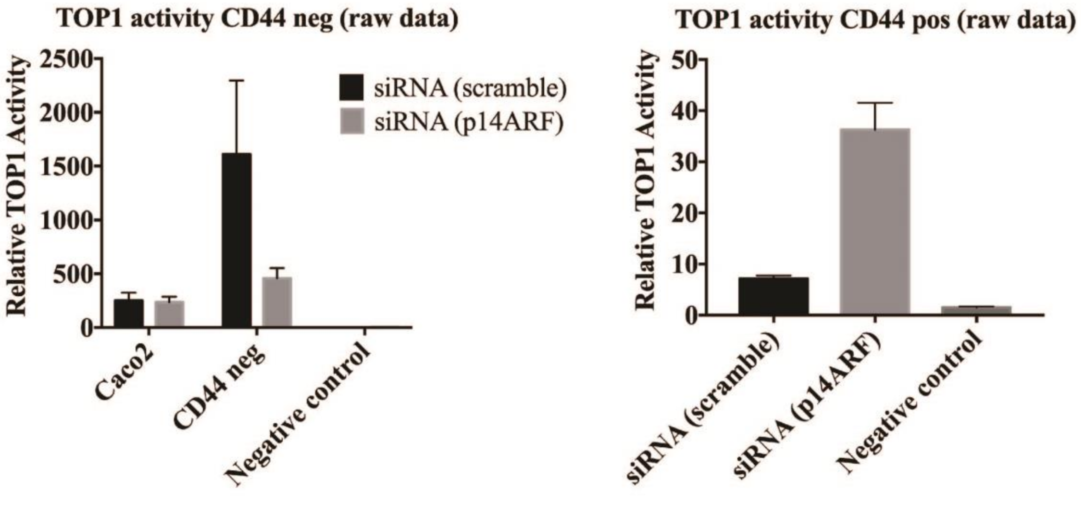

Figure S7. TOP1 activity with or without downregulation of p14ARF in the Caco2, non-CSC-like and CSC-like cells**.** Left graph, measurement of TOP1 activity in the whole cell extracts from Caco2 or

Caco2 non-CSC-like (CD44 neg) cells transfected with siRNA (scramble) (black bars) or siRNA (p14ARF) (grey bars). The CD44 negative cells were obtained by NaBt treatment giving approx. 100% cell differentiation (Figure 1A). TOP1 activity was measured by the REAAD assay, and the REEAD signal quantified by ImageJ software and plotted as mean +/− SEM. Right graph, measurement of TOP1 activity in the whole cell extracts from Caco2 CSC-like (CD44 pos) cells transfected with siRNA (scramble) (black bars) or siRNA (p14ARF) (grey bars). The CD44 positive cells were captured onto a glass slide by using anti CD44 antibody. Following cell lysis, the TOP1 activity was measured by using the On-Slide-REEAD assay as described in [13]. The REEAD signals were quantified by ImageJ software and plotted as mean +/− SEM. In both experiments the number of signals counted were normalized to the number of signals obtained when analyzing a standard sample containing circularized substrate obtained by incubation with purified TOP1. Hence, the results are shown as TOP1 activity relative to the standard. This was done to avoid experimental noise resulting from slide-to-slide variations as previously described [1,12,14].

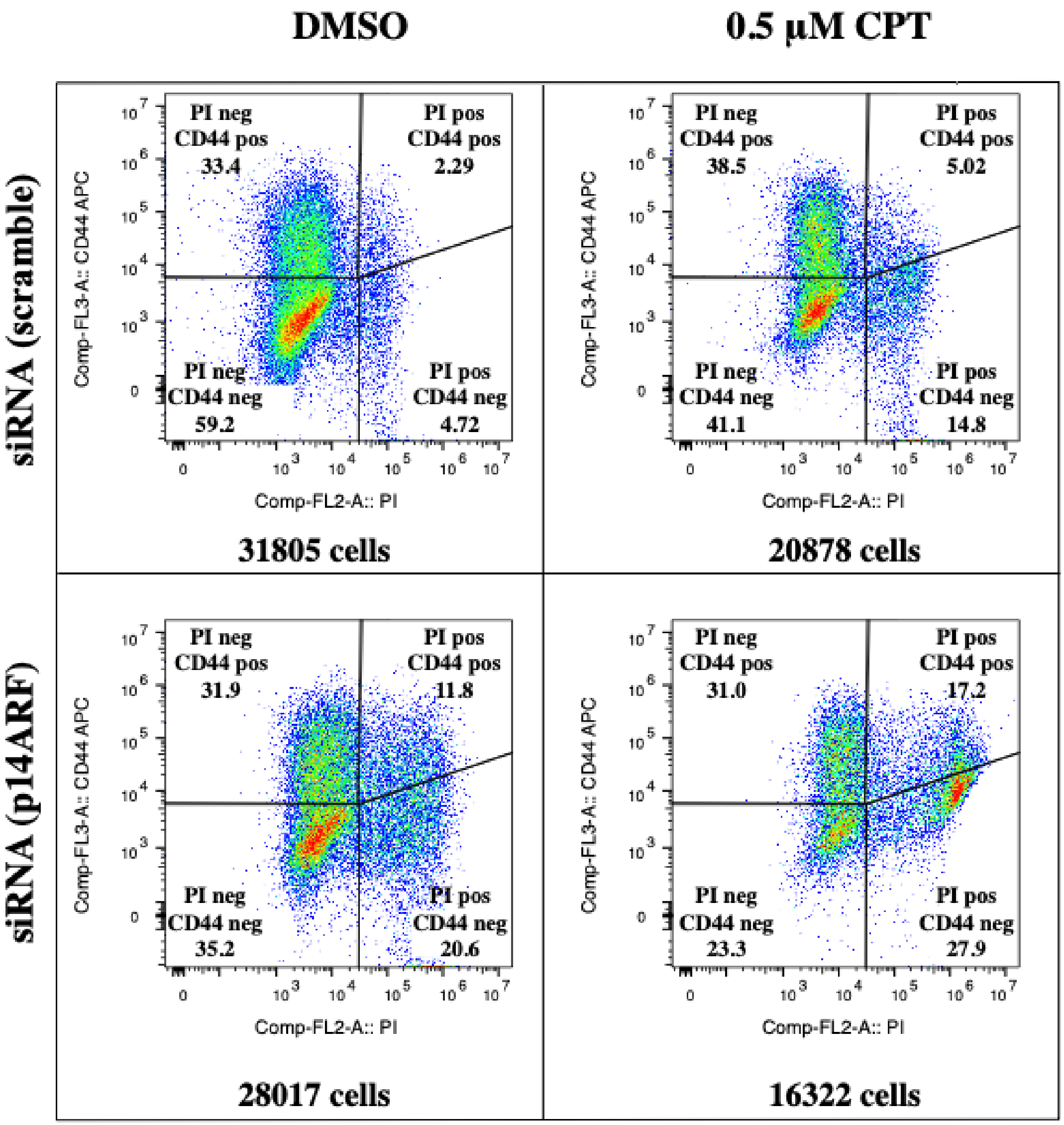

Figure S8. Effect of the p14ARF downregulation on the non-CSC-like and CSC-like CPT sensitivity. Caco2 cells were transfected with siRNA (scramble) (upper panels) or siRNA (p14ARF) (lower panels) and treated with DMSO (left panels) or 0.5 μM CPT. Cells were harvested and labelled with APC conjugated anti CD44 antibody and stained with propidium iodide (PI) before they were analyzed by

flow-cytometry using the Beckman Cytoflex instrument. Data were analyzed using FlowJo software and compensated by using single stained samples. The dot-plots show compensated PI values (Comp-FL2-A:PI, x-axis) versus compensated CD44-APC values (Comp-FL3:CD44 APC, y-axis). In each panel the total number of analyzed cells is reported at the bottom of the dot plots.

Supplementary S9

p14ARF Knockdown Prevents Accumulation of Cancer Stem Cell-like Caco2 Cells Upon Camptothecin Treatment.

The enhanced TOP1 activity in CSC cells after p14ARF knockdown suggests that reducing the p14ARF expression may enhance the CPT response of these cells. To investigate this possibility Caco2 cells were treated with scrambled or p14ARF specific siRNA before they were treated with 0.5 µM CPT or DMSO. After 72 hours, the percentages of CSC-like or non-CSC-like cells in the surviving population were determined by flow-cytometry analyses. Similar to the observations shown in Figure 2 the size of the CSC-like CD44 positive cell fraction increased markedly from 27% to 57% in cells treated with scrambled siRNA and CPT (Supplementary Figure S9) confirming the CPT resistance of the CSC-like cells. This changed dramatically upon p14ARF knockdown leading to a rather modest increase in the percentage (from 25% to 34%) of CSC-like cells upon CPT treatment (Supplementary Figure S9, bottom panels). This strongly indicates an increased CPT response of the CSC-like cell subpopulation upon p14ARF knockdown and suggests that CPT resistance of CSC-like cells may be counteracted by p14ARF knockdown. When considering treatment, it is of outmost importance to eradicate in particular the CSC-like cells since numerous studies have shown that only those possess tumorigenic capacity [15].

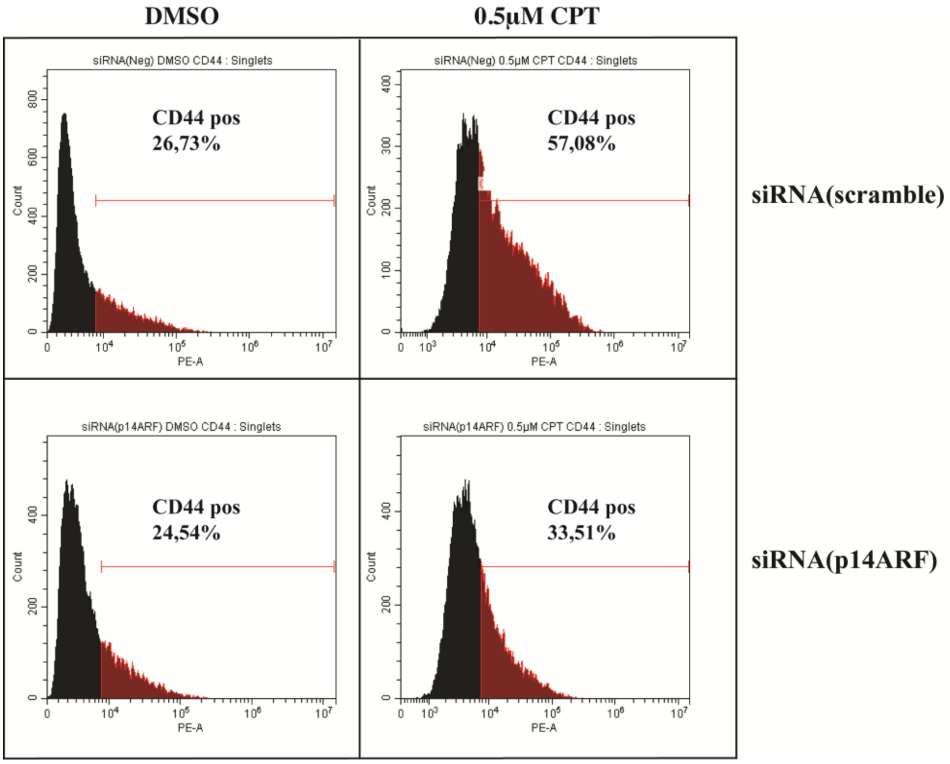

Figure S9. p14ARF Knockdown Prevents Accumulation of CSC-like Caco2 Cells Upon CPT treatment. The Caco2 cells were transfected with siRNA (scramble) (top panels) or siRNA (p14ARF) (bottom panels) and treated with DMSO (left panels) or 0.5 µM CPT (right panels) for 72 hours before they were stained with PE conjugated anti CD44 antibody. The cells were analyzed by flow-cytometry using the Beckman Cytoflex instrument. Cell debris and doublet were removed from the analysis using FSC and SSC gatings. The percentages CSC-like Caco2 cells (CD44 pos) are shown in red.

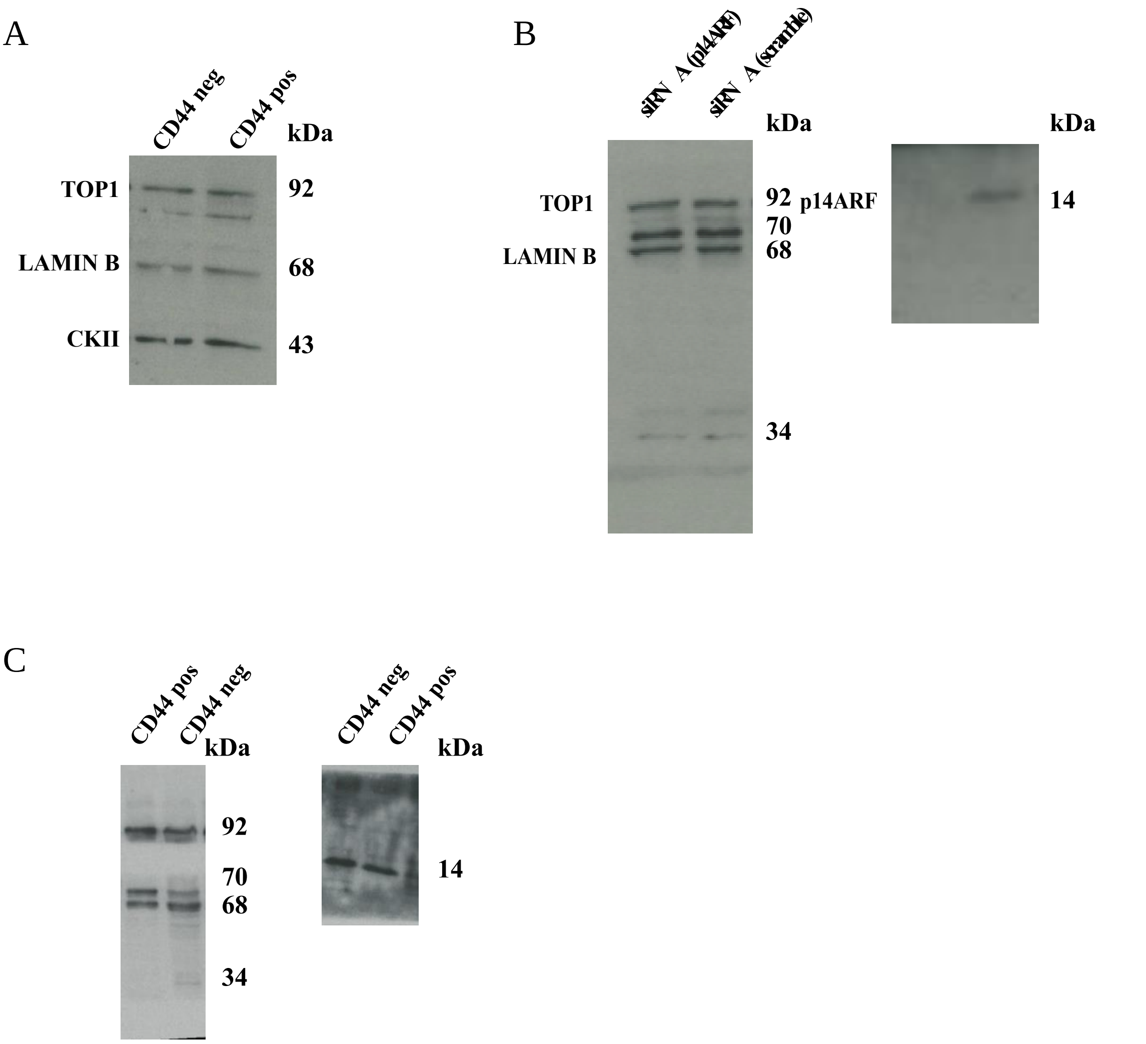

Figure S10. Original blots. (**A**) Original blot of the western blot shown in Figure 3A. (**B**) Original blot of the western blots shown in Figure 4A. (**C**) Original blot of the western blot shown in Supplementary Figure S6A. For all blots Molecular weights are indicated at the right of the blots.

Supplementary Materials and Methods

CKII Activity

The activity of CKII in Caco2 and DLD1 nuclear extracts was measured using the Millipore Casein Kinase 2 Assay Kit (#17-132). The GST tagged N-terminal domain of TOP1 (a.a. 1-206) (p25) was used as substrate and purified as described previously [16]. Nuclear extracts from 10^7 Caco2 or DLD1 cells treated with DMSO or 10 µM TBB were prepared as described in the material and method section of the main text and incubated with the substrate with the buffers provided by the kit and 12.5 mCurie/ml α^{32}P-dATP. The reactions were incubated at 30°C for different time intervals and the reactions stopped with addition of 0.5% SDS. The proteins were run on a 10% SDS gel in 25 mM Tris-HCl pH 8.6, 192 mM glycine, 0.1% SDS for 1 hour at 50 mA constant. The proteins were transferred onto a nitrocellulose membrane using a wet blotting apparatus for 16 hours at 30 V constant in a 20 mM CAPS pH 10 and 20% Ethanol at 4°C. The membranes were exposed in a PhosphorImager cassette for 16 hours. The intensities of the radioactive bands were quantified using QuantityOne software and normalized to the highest intensity of the bands arising from the DMSO treated cells at 20 and 40 minutes for the experiments with DLD1 and Caco2, respectively. The membranes were stained with Ponceau to check that the amount of p25 substrate was the same in all the reactions. The

nuclear extracts used were normalized using Bradford quantification. The results were plotted as mean +/- SEM

De-phosphorylation of Topoisomerase 1

De-phosphorylation was performed by incubation of cellular extracts from either total or FACS sorted CD44 neg, CD44 pos, CD133 negative and CD133 positive Caco2 and DLD1 cells, respectively. The cells were subjected to de-phosphorylation by treatment with the lambda protein phosphatase (λPpase) (1600 units) in λPpase buffer at 30°C for 45 minutes according to manufacturer's instructions (New England, BioLabs. Inc, USA). The identical aliquot of each cell fraction was mock treated as a control in parallel. The reaction was stopped by freezing the samples at -80°C followed by lyophilization. The samples were then solubilized in CLB1 lysis buffer (R&D Systems, Inc, USA) and subjected either to one or two-dimensional polyacrylamide electrophoresis (2D PAGE) followed by Western blot.

The efficiency of phosphatase treatment was controlled by probing the blot where MCF7 breast cancer cells transformed with the activated form of Δp95 ERbB2 protein were separated with phosphor-specific antibodies against Erk1/2 (data not shown).

SDS-PAGE, 2D PAGE and Western blot

The separation of proteins by one-dimensional SDS-PAGE gel was performed by using NuPAGE® system (Invitrogen A/S, Denmark) according to manufacturer's instructions. In brief, cell lysates were mixed with NuPAGE®LDS loading buffer and NuPAGE® sample reducing agent. Samples were then incubated at 70°C for 10 min and loaded onto NuPAGE® Novex 4-12% Bis-Tris gels with 50 μg/lane (10^4 cells). The protein separation was performed in NuPAGE® MOPS buffer containing NuPAGE®antioxidant according to manufacturer's instructions.

The 2D PAGE was carried out using an immobilized pH gradient (IPG) as previously described [17]. Briefly, the IPG strips pI 3-10, were actively rehydrated with 200 μl of sample solution containing proteins from approximately $0.5x10^6$ cells. The isoelectrofocusing was carried out for 18,000 Vhr. using the Bio-Rad PROTEAN IEF (Bio-Rad laboratories, Denmark). The second dimension was carried out as previously described [18]. All samples were run twice.

Western blot was performed according to procedure described elsewhere [18]. The resolved proteins were blotted onto Hybond™-C nitrocellulose membranes (Amersham Biosciences, USA) and reacted with a TOP1 specific rabbit antibody (1:2000; Epitomics, USA) followed by detection of immune complexes with a horseradish peroxidase-labeled polymer (1:200) (Envision+ detection kit; DAKO; Denmark). The blots were developed using the Amersham ECL™ plus Western Blotting Detection Kit (GE Healthcare/Amersham Bioscience, VWR, Denmark) according to manufacturer's instructions. The membranes were additionally reversibly stained with Ponceau S solution (Sigma-Aldrich, Denmark) to match location of proteins in membrane with the Western blot signal and to ensure a proper focusing of protein spots. As a loading control the blots were developed with mouse monoclonal anti alpha-tubulin antibodies (1:2000; Santa Cruz, USA).

Flow-Cytometry Analysis

For flow-cytometry analysis, the Caco2 were harvested by trypsin treatment, 0.25% trypsin, 0.02% EDTA, (Sigma-Aldrich ApS, Denmark). After re-suspension of the cells in cell-culture media the cells were counted using a Bürker-Türk chamber (Sigma-Aldrich, Denmark A/S), washed in PBS with 0.5% BSA and collected by centrifugation. The cells were incubated for 30 minutes in blocking buffer (1xPBS, 1% BSA, 2.5 mM EDTA, 25 mM HEPES, 20% FcR blocking reagent (MACS Miltenyi Biotech)) at 4°C, and stained for 30 minutes at 4°C with APC conjugated CD44 (MACS Miltenyi Biotech) following the manufacturer's instructions. Following the antibody labeling, the cells were washed in PBS with 0.5% BSA before flow-cytometric analysis that was carried out on a Cytoflex (Beckman Coulter) instrument. Dying and dead cells were stained with 1 μg/ml propidium iodide (PI). APC and PI were excited with a 638 or 561 nm laser, respectively, and fluorescence emitted was

collected with a 660/20 or 585/42 band pass filter, respectively. Doublets and cell debris were excluded by gating with FSC and SSC. CytExpert software (Beckman Coulter) was used to perform the analysis. Gates were generated based on unstained cells and single stained cells.

References

1. Roy, A.; Tesauro, C.; Frøhlich, R.; Hede, M.S.; Nielsen, M.J.; Kjeldsen, E.; Bonven, B.; Stougaard, M.; Gromova, I.; Knudsen, B.R. Decreased camptothecin sensitivity of the stem-cell-like fraction of Caco2 cells correlates with an altered phosphorylation pattern of topoisomerase I. *PLoS One* **2014**, *9*, e99628, doi:10.1371/journal.pone.0099628.
2. Dalerba, P.; Dylla, S.J.; Park, I.-K.; Liu, R.; Wang, X.; Cho, R.W.; Hoey, T.; Gurney, A.; Huang, E.H.; Simeone, D.M.; et al. Phenotypic characterization of human colorectal cancer stem cells. *Proc. Natl. Acad. Sci. U. S. A.* **2007**, *104*, 10158–63, doi:10.1073/pnas.0703478104.
3. Haraguchi, N.; Ohkuma, M.; Sakashita, H.; Matsuzaki, S.; Tanaka, F.; Mimori, K.; Kamohara, Y.; Inoue, H.; Mori, M. CD133+CD44+ population efficiently enriches colon cancer initiating cells. *Ann. Surg. Oncol.* **2008**, *15*, 2927–2933, doi:10.1245/s10434-008-0074-0.
4. Ferrand, A.; Sandrin, M.S.; Shulkes, A.; Baldwin, G.S. Expression of gastrin precursors by CD133-positive colorectal cancer cells is crucial for tumour growth. *Biochim. Biophys. Acta - Mol. Cell Res.* **2009**, *1793*, 477–488, doi:10.1016/j.bbamcr.2009.01.004.
5. Chen, K.L.; Pan, F.; Jiang, H.; Chen, J.F.; Pei, L.; Xie, F.W.; Liang, H.J. Highly enriched CD133 +CD44 + stem-like cells with CD133 +CD44 high metastatic subset in HCT116 colon cancer cells. *Clin. Exp. Metastasis* **2011**, *28*, 751–763, doi:10.1007/s10585-011-9407-7.
6. Gupta, P.B.; Fillmore, C.M.; Jiang, G.; Shapira, S.D.; Tao, K.; Kuperwasser, C.; Lander, E.S. Stochastic state transitions give rise to phenotypic equilibrium in populations of cancer cells. *Cell* **2011**, *146*, 633–644, doi:10.1016/j.cell.2011.07.026.
7. Du, L.; Wang, H.; He, L.; Zhang, J.; Ni, B.; Wang, X.; Jin, H.; Cahuzac, N.; Mehrpour, M.; Lu, Y.; et al. CD44 is of functional importance for colorectal cancer stem cells. *Clin. Cancer Res.* **2008**, *14*, 6751–6760, doi:10.1158/1078-0432.CCR-08-1034.
8. Wang, C.; Xie, J.; Guo, J.; Manning, H.C.; Gore, J.C.; Guo, N. Evaluation of CD44 and CD133 as cancer stem cell markers for colorectal cancer. *Oncol. Rep.* **2012**, *28*, 1301–8, doi:10.3892/or.2012.1951.
9. Clevers, H. Stem cells, asymmetric division and cancer. *Nat. Genet.* **2005**, *37*, 1027–1028, doi:10.1038/ng1005-1027.
10. Tirino, V.; Desiderio, V.; Paino, F.; Papaccio, G.; Rosa, M. De Methods for Cancer Stem Cell Detection an isolation. *Methods Mol. Biol.* **2012**, *879*, 513–529, doi:10.1007/978-1-61779-815-3.
11. Pine, S.R.; Liu, W. Asymmetric cell division and template DNA co-segregation in cancer stem cells. *Front. Oncol.* **2014**, *4*, 226, doi:10.3389/fonc.2014.00226.
12. Stougaard, M.; Lohmann, J.; Mancino, A.; Celik, S.; Andersen, F.F.; Koch, J.; Knudsen, B.R. Single-Molecule Detection of Human Topoisomerase I Cleavage− Ligation Activity. *ACS Nano* **2008**, *3*, 223–233.
13. Keller, J.G.; Tesauro, C.; Coletta, A.; Graversen, A.D.; Ho, Y.-P.; Kristensen, P.; Stougaard, M.; Knudsen, B.R. On-slide detection of enzymatic activities in selected single cells. *Nanoscale* **2017**, *9*, doi:10.1039/c7nr05125e.
14. Andersen, F.F.; Stougaard, M.; Jørgensen, H.L.; Bendsen, S.; Juul, S.; Hald, K.; Andersen, A.H.; Koch, J.; Knudsen, B.R. Multiplexed detection of site specific recombinase and DNA topoisomerase activities at the single molecule level. *ACS Nano* **2009**, *3*, 4043–54, doi:10.1021/nn9012912.
15. O'Brien, C. a; Pollett, A.; Gallinger, S.; Dick, J.E. A human colon cancer cell capable of initiating tumour growth in immunodeficient mice. *Nature* **2007**, *445*, 106–10, doi:10.1038/nature05372.
16. Lisby, M.; Olesen, J.R.; Skouboe, C.; Krogh, B.O.; Straub, T.; Boege, F.; Velmurugan, S.; Martensen, P.M.; Andersen, A.H.; Jayaram, M.; et al. Residues Within the N-terminal Domain of Human Topoisomerase I Play a Direct Role in Relaxation. *J. Biol. Chem.* **2001**, *276*, 20220–20227, doi:10.1074/jbc.M010991200.
17. Gromov, P.; Celis, J.E.; Gromova, I.; Rank, F.; Timmermans-Wielenga, V.; Moreira, J.M.A. A single lysis solution for the analysis of tissue samples by different proteomic technologies. *Mol. Oncol.* **2008**, *2*, 368–79, doi:10.1016/j.molonc.2008.09.003.
18. Celis, J.E.; Trentemølle, S.; Gromov, P. High-Resolution Two-Dimensional Gel Electrophoresis of Proteins. Isoelectric Focusing and Nonequilibrium pH Gradient Electrophoresis. In *Cell Biology*; 2006; pp. 165–174 ISBN 9780121647308.
